# Deep Sequencing Discovery and Profiling of Known and Novel miRNAs Produced in Response to DNA Damage in Rice

**DOI:** 10.3390/ijms22189958

**Published:** 2021-09-15

**Authors:** Jianxiang Zhang, Cheng Xu, Kangwei Liu, Yaoqinq Li, Mengna Wang, Lian Tao, Hengxiu Yu, Chao Zhang

**Affiliations:** Key Laboratory of Plant Functional Genomics of the Ministry of Education/Jiangsu Key Laboratory of Crop Genomics and Molecular Breeding/Jiangsu Key Laboratory of Crop Genetics and Physiology/Joint International Research Laboratory of Agriculture and Agri-Product Safety, The Ministry of Education of China/Jiangsu Co-Innovation Center for Modern Production Technology of Grain Crops, Agricultural College of Yangzhou University, Yangzhou 225009, China; DX120170073@yzu.edu.cn (J.Z.); MZ120190944@yzu.edu.cn (C.X.); DX120200117@yzu.edu.cn (K.L.); 191702406@yzu.edu.cn (Y.L.); MX120200748@yzu.edu.cn (M.W.); MZ120201197@yzu.edu.cn (L.T.)

**Keywords:** rice, DNA damage repair, transcriptome-level sequencing, miRNA, RT-qPCR

## Abstract

Under extreme environmental conditions such as ultraviolet and ionizing radiation, plants may suffer DNA damage. If these damages are not repaired accurately and rapidly, they may lead to chromosomal abnormalities or even cell death. Therefore, organisms have evolved various DNA repair mechanisms to cope with DNA damage which include gene transcription and post-translational regulation. MicroRNA (miRNA) is a type of non-coding single-stranded RNA molecule encoded by endogenous genes. They can promote DNA damage repair by regulating target gene transcription. Here, roots from seedlings of the *japonica* rice cultivar ‘Yandao 8’ that were treated with bleomycin were collected for transcriptome-level sequencing, using non-treated roots as controls. A total of 14,716,232 and 17,369,981 reads mapping to miRNAs were identified in bleomycin-treated and control groups, respectively, including 513 known and 72 novel miRNAs. Compared with the control group, 150 miRNAs showed differential expression levels. Target predictions of these differentially expressed miRNAs yielded 8731 potential gene targets. KEGG annotation and a gene ontology analysis indicated that the highest-ranked target genes were classified into metabolic processes, RNA degradation, DNA repair, and so on. Notably, the DNA repair process was significantly enriched in both analyses. Among these differentially expressed miRNAs, 58 miRNAs and 41 corresponding potential target genes were predicted to be related to DNA repair. RT-qPCR results confirmed that the expression patterns of 20 selected miRNAs were similar to those from the sequencing results, whereas four miRNAs gave opposite results. The opposing expression patterns of several miRNAs with regards to their target genes relating to the DNA repair process were also validated by RT-qPCR. These findings provide valuable information for further functional studies of miRNA involvement in DNA damage repair in rice.

## 1. Introduction

There are many environmental factors that can cause DNA damage in living organisms, such as ultraviolet, ionizing radiation, alkylating agents, base analogs, and many more. If DNA damage is not rapidly and accurately repaired, it can lead to permanent genetic changes that significantly alter the organisms’ genetic and phenotypic characteristics [1,2]. Extensive DNA damage can even cause chromosomal instabilities resulting in the death of cells and/or the whole organism [3]. Hundreds of genes related to DNA damage repair have been identified. They are primarily involved in five different, but functionally related, pathways: base excision repair (BER), nucleotide excision repair (nucleotide excision repair, NER), mismatch repair (MMR), non-homologous end joining (NHEJ), and homologous recombination (HR) [4]. DNA double-strand breaks (DSBs) are one type of DNA damage that can cause deleterious genetic lesions that drive genetic instability. DSB repair is crucial to preserving genome integrity in all living organisms. NHEJ and HR are the two major repair mechanisms involved in repairing DSBs [5]. NHEJ promotes direct ligation of the DSB ends and is an efficient but error-prone manner, while HR is largely error free, but it requires homologous DNA sequences as a template for repair by DNA synthesis [6,7].

Micro RNAs (miRNAs) are a type of small (20-24 nucleotide), non-coding, single-stranded RNA molecule that are encoded by endogenous genes and involved in post-transcriptional gene regulation [8,9]. Similar to messenger RNA (mRNA), an intact miRNA is synthesized by DNA-dependent RNA polymerase II (Pol II) and must undergo a series of co-transcriptional modifications before maturation, including 5′ end capping, and 3′ end polyadenylation or splicing. In plants, miRNAs play important roles in physiological processes, especially in gene regulatory networks [10,11,12]. Many miRNA gene targets, such as those that encode transcription factors, are self-regulated [13,14]. Additionally, many non-transcription factor gene targets encode F-box proteins or ubiquitin-conjugating enzymes, which are involved with proteasome degradation, suggesting that miRNAs also play a role in regulating protein stability and plant development [15,16]. For example, the *DOG1* gene regulates the dormancy and flowering time of Arabidopsis through the miRNA 156/172 pathway [17]; many miRNAs in *Brassica napus* participate in regulating flowering time [18]. MPeARF17.2 participates in the regulation of adventitious root development in poplar trees by regulating hormone synthesis [19]; about 28 miRNAs participate in the low-temperature stress response of *alfalfa* standard varieties and regulate the fall and winter dormancy processes [20].

Increased scientific interest in miRNA functions has revealed that miRNAs play important roles in abiotic stress responses including drought, cold, nutrient deficiency, oxidative stress, submergence, UV-B radiation, and virus infection [21,22,23]. One common trait shared between these various abiotic stressors is that they all induce an oxidative burst with damaging effects on cellular macromolecules such as lipids, enzymes, and DNA, of which DNA damage is the most serious [24,25,26,27,28,29,30]. Plants have evolved a series of reliable mechanisms to deal with DNA damage, and the miRNA-based regulation mechanism is the more common among them. For instance, miR4-MP and miR4-Rep can target Cucumber Green Mottle Mosaic Virus (CGMMV) to reduce DNA damage and depress damage in *N. nicotiana* leaves by reducing CGMMV replication [31]. Furthermore, miR529a alleviates DNA damage by regulating *OsSPL* family genes [32]. Many miRNAs involved in the DNA repair process have been identified in *Arabidopsis* [33]. However, due to the limitations of materials and no concentrated research on target genes related to DNA repair, the mechanism of miRNAs regulating DNA damage repair remains unclear.

In this study, we use bleomycin to simulate extreme environmental stress to induce DNA damage in rice seedling roots. Differentially expressed miRNAs were identified by high-throughput sequencing of bleomycin-treated and non-treated control groups. The corresponding target genes of differentially expressed miRNAs were predicted. Additionally, the expression patterns of several miRNAs and their target genes relating to DNA repair were validated by RT-qPCR. Our study suggests that miRNAs may be widely involved in regulating DNA damage repair in rice and provides novel insights into the molecular mechanisms of DNA damage repair.

## 2. Results

### 2.1. DNA Damage Induction by Bleomycin

**Figure 1:**
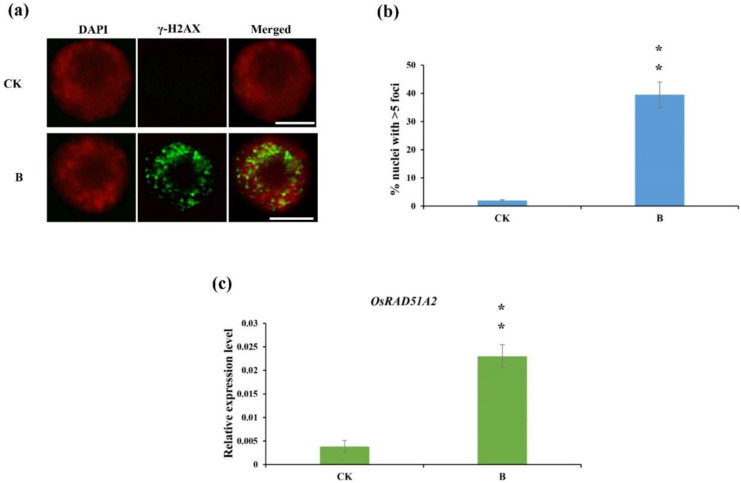
Immunostaining of γ-H2AX and *OsRAD51A2* protein expression in rice roots. (**a**) Immunostaining of γ-H2AX (green) in rice root tip cells treated with bleomycin at concentrations of 0 (CK) or 10 (B) μg/mL for 3 h. Chromosomes are stained with DAPI (red). Scale bar, 10 μm; (**b**) Quantification of γ-H2AX immune signals in (**a**). Quantification of the nuclei containing γH2AX focus signals was obtained by counting at least 100 interphase nuclei, and scoring ≥50 foci as positive. Data represent the means and standard deviations (SDs) of the results of three independent experiments. Student’s t-test: ** *p <* 0.01; (**c**) RT-qPCR analysis of *OsRAD51A2* expression in rice roots treated with bleomycin at concentrations of 0 (CK) or 10 (B) μg/mL for 24 h. Data represent the means ± SDs of three replicates. Student’s t-test: ** *p <* 0.01.

To better understand rice miRNA expression in response to DNA damage, DNA double-strand breaks (DSBs) were induced in rice seedling roots via treatment with the DNA damage inducer bleomycin for 3 h, after which the DNA damage response (DDR) was determined. γH2AX foci are considered to be reliable markers of DSBs [34,35]. To investigate whether DSBs occurred after bleomycin treatment, antibodies that recognize γH2AX were used in immunostaining. Many γH2AX foci signals (42.5 ± 11.8%) were observed in the interphase nuclei of root tip cells treated with bleomycin, while almost no γ-H2AX signal (3.4 ± 1.1%) was observed in the control group cells (Figure 1a,b). This suggests that bleomycin induces DNA damage in rice seedlings.

Our previous study shows that *RAD51A2* is involved in DNA repair and sensitive to DNA damage at the transcriptional level [36]. To analyze whether a DDR event can be trigged by bleomycin treatment, we also treated rice roots with bleomycin for 24 h and analyzed the expression level of the *RAD51A2* gene by RT-qPCR. The transcription level of the *RAD51A2* gene in bleomycin-treated roots was about five-fold higher than that of untreated control roots (Figure 1c). These results indicated that DNA damage and DNA repair were indeed triggered in rice roots after bleomycin treatment.

### 2.2. The Effects of Bleomycin on Rice Growth

**Figure 2:**
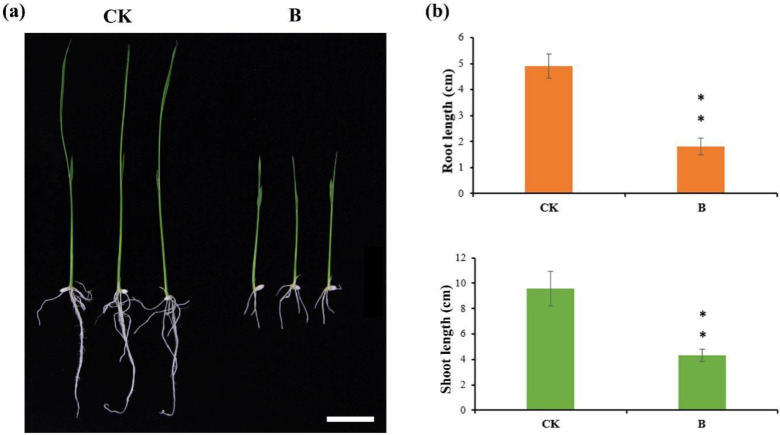
Phenotype of seedlings treated with bleomycin. (**a**) Morphological comparison of seedlings treated with 0 (left) or 10 μg/mL (right) bleomycin for 12 d. Scale bar = 2 cm; (**b**) Length of seedling roots (above) and shoots (below) treated with bleomycin at concentrations of 0 (CK) or 10 (B) μg/mL for 12 d. Student’s t-test: ** *p <* 0.01.

DNA DSBs are among the most serious types of damage to cells, and can result in the loss and rearrangement of genetic information that leads to mutation or cell death [3]. In order to study the performance of rice treated with bleomycin, sterilized seeds were incubated on a solid MS medium containing 0 or 10 μg/mL bleomycin for 12 days. As predicted, significantly different phenotypes were observed between the two groups (Figure 2a). The seedlings treated with 10 μg/mL bleomycin had significantly shorter roots and shoots than the seedlings in the control group (Figure 2b). This shows that bleomycin treatment has an inhibitory effect on rice seedling growth.

### 2.3. Small RNAs Identified by High-Throughput Sequencing

**Figure 3:**
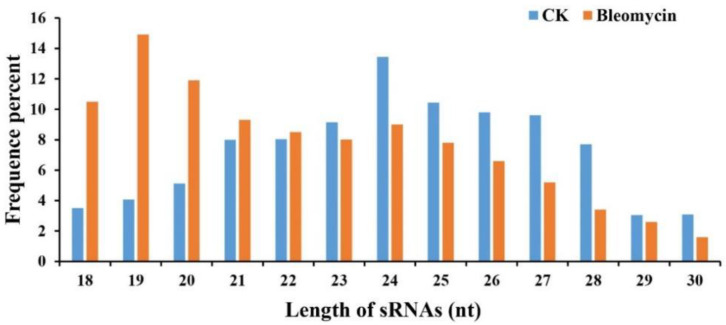
The length distribution of total sRNAs from different groups. Blue box and bars represent the CK (non-treated control) treatment; orange box and bars represent the bleomycin treatment (bleomycin). Y-axis depicts the proportion of reads identified among total reads. X-axis depicts the length of small RNAs.

To identify rice miRNAs involved in the DDR process, two groups of RNA libraries were constructed from rice roots treated with bleomycin or left untreated and sequenced with Illumina HiSeq2000 technology, producing 14,716,232 and 17,369,981 raw reads, respectively (Table 1). After removing low-quality reads, poly A/T/G/C reads, adaptor sequences, and N% > 10% reads, an average of 97.54% of the total reads remained. A total of 14,248,017 and 17,091,722 clean reads were obtained for the bleomycin and control groups, respectively (Table 1). After filtering out reads without small RNA sequences, we obtained and analyzed those small RNAs in the 18–30 nt size range (Figure 3). The most abundant small RNAs in the control and bleomycin treatment libraries were 24 nt and 19 nt in length, respectively. The distribution of 24 nt small RNAs was approximately 13.45% and 8.99% in the control and bleomycin treatment libraries, respectively, while the distribution of 19 nt small RNAs was approximately 4.07% and 14.92% in the control and bleomycin treatment libraries, respectively. A total of 4,736,578 (bleomycin treatment) and 12,819,498 (control) small RNA sequences were successfully mapped to the rice reference genome, of which the mapped small RNAs were clustered into several RNA classes including known miRNAs, rRNAs, tRNAs, snRNAs, snoRNAs, repeats, natural antisense transcripts, and novel miRNAs (Table 2). In the control library, 246,484 and 1629 reads were identified as putative known and novel miRNAs, respectively. In the bleomycin treatment library, 28,315 and 284 reads were identified as putative known and novel miRNAs, respectively.

### 2.4. Identification of Known and Novel miRNAs Relating to DDR for Rice

**Figure 4:**
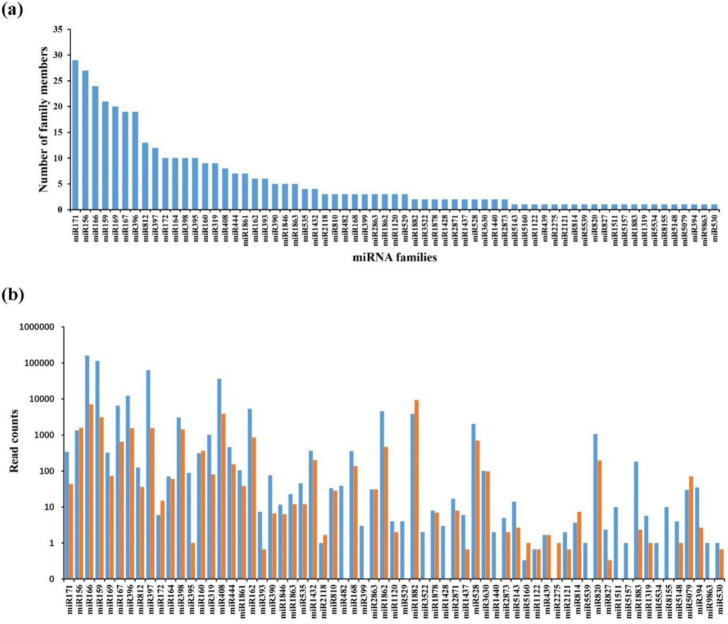
Known miRNAs in rice. (**a**) Known miRNAs and their family numbers, in rice roots. Y-axis depicts the number of members identified in each miRNA family. X-axis shows the 65 known miRNA families identified in the two libraries; (**b**) Read counts of the 65 known miRNA families in the two libraries.

To identify miRNAs produced in response to the DNA damage in rice, we screened 13,340 and 959 unique sRNAs from the identified known and novel miRNAs, respectively. The unique sRNAs were mapped to known mature plant miRNAs from the miRBase database and 512 mature known miRNAs were identified in two libraries: 468 and 371 in the control and bleomycin treatment libraries, respectively (Table S1). Among these miRNAs, 327 known miRNAs (64%) were shared between the two libraries, while 186 (36%) miRNAs were detected in one library, implying vastly different miRNA components in the two libraries (Table S1). These 367 known miRNAs belonged to different miRNA families (Table 3). The number of members in different miRNA families are listed in Table 3. It shows various numbers of miRNA members in different miRNA families; miR171 was the most represented family with 29 members, followed by miR156, miR166, miR159, and miR169, with 27, 24, 21, and 20 members, respectively. As listed in Figure 4a, eight families, including miR167, miR396, miR812, miR397, miR172, miR164, miR398, and miR395 had more than 10 miRNAs each. The rest of the 52 families had few miRNAs, with less than 10 members and there were 145 known miRNAs with no match to any family, possibly due to the variation between the rice species used and that of the reference genome (Table S1).

We further analyzed the expression levels of the known miRNA families and found great differences in the read counts with miR166, miR159, miR397, and miR408 representing the top four families in the control libraries and miR1882, miR166, miR408, and miR159 in the bleomycin treatment libraries (Figure 4b). The miR166 family was represented the most frequently, with 159,766 and 7058 read counts in the control and bleomycin treatment libraries, respectively. Furthermore, miR156, miR160, miR164, and miR3630 were moderately abundant in the two libraries, while several miRNAs such as miR399, miR529, miR2275, miR5160 were absent from one of the two libraries (Figure 4b). This indicated that the miRNAs presented in the control library are different from those in the bleomycin treatment library.

To identify novel miRNA candidates in the control and bleomycin treatment libraries, the 13,599,747 and 5,088,932 sequences lacking annotation were mapped against the rice mRNA transcriptome database, respectively. The formation of a stable hairpin structure is a prerequisite for annotating novel miRNAs. In total, 72 potential novel miRNAs were predicted from the two libraries (Table S2). The stem loop structures of these predicted miRNA precursors are shown in Figure S1. Twenty-three of the 72 novel miRNA candidates existed in one library, and 49 out of the 72 novel miRNA candidates were shared by the two libraries. We found that 72 novel miRNAs were all lowly expressed (<1000 read counts) in the two libraries (Table S2). Additionally, it is reported that the existence of complementary sequences increases the authenticity of predicted novel miRNAs [37]. In this study, 42 of the 72 novel miRNAs had complementary miRNAs.

### 2.5. Differentially Expressed miRNAs

**Figure 5:**
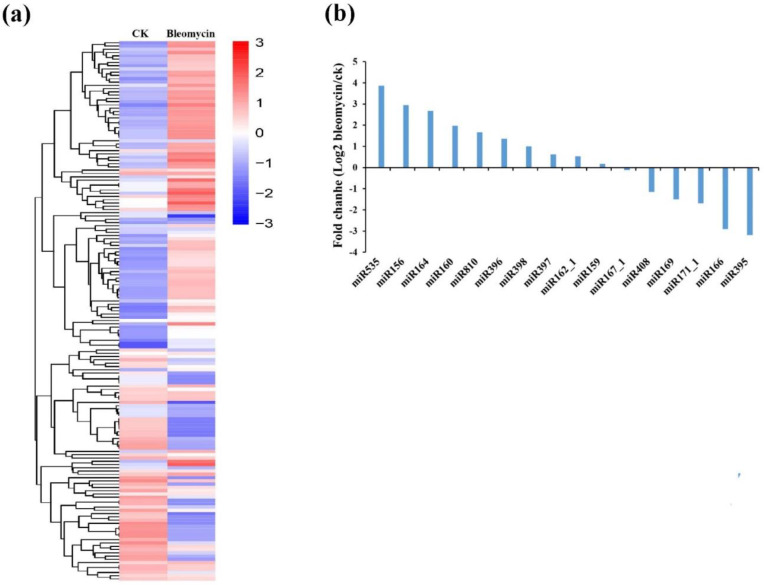
The differentially expressed miRNAs and conserved miRNA families in the two libraries. (**a**) Hierarchical cluster and expression profile analyses of the differentially expressed miRNAs between the CK (control) and bleomycin treatment libraries (Bleomycin). Each column represents a sample, and each row represents a single miRNA. The data in the heatmap are the log10 values of transcripts per million (TPM). The bar indicates the expression scale for miRNAs; Red represents highly expressed miRNAs and grey represents non-expressed miRNAs; (**b**) A comparison of the relative expression of differentially expressed conserved miRNA families. The ordinate is the difference multiple of each differentially expressed miRNA (log_2_ Bleomycin/CK). The abscissa represents differentially expressed conserved miRNA families.

The normalized expressions of miRNAs in the CK and bleomycin treatment groups were compared. Based on the selected criteria (|log2 fold-change| > 1, FDR < 0.05), 140 known and 10 novel miRNAs were identified as differentially expressed (Table S3). We used clustering to show the difference in miRNA expression between the bleomycin treatment and control groups (Figure 5a). Compared with the control library, 69 miRNAs were significantly up-regulated including hci-miR156a, stu-miR156f-5p, cca-miR156b, and osa-miR1860-5p, while 81 miRNAs were down-regulated including gma-miR166m, ppt-miR166j, osa-miR166k-3p, and osa-miR1883a in the bleomycin treatment library. These results indicate that the bleomycin reagent can affect the expression of some miRNAs. A total of 16 differentially expressed conserved miRNA families were obtained from the 65 known miRNA families (Figure 4a). Among the 16 conserved miRNA families, ten and six miRNA families were either up- or down-regulated, respectively, after bleomycin treatment (Figure 5b). The difference in expression profiles suggests a relationship between miRNA expression and bleomycin-induced DNA damage repair.

### 2.6. Identification of Potential Gene Targets of Differentially Expressed miRNAs

**Figure 6:**
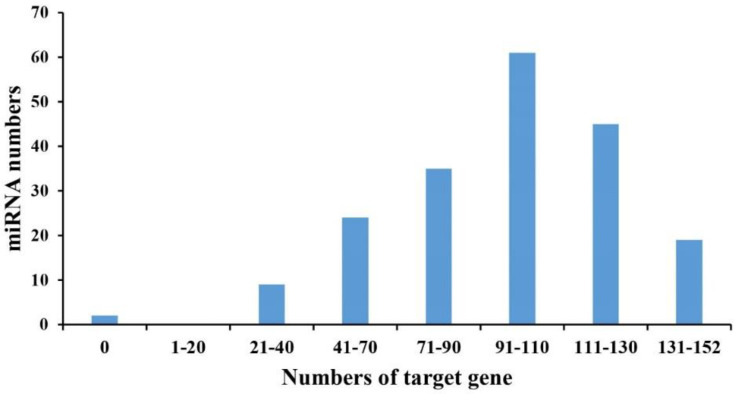
The distribution of target gene numbers for differentially expressed miRNAs. X-axis depicts the interval for the target gene number of each miRNA. Y-axis depicts the number of miRNAs in each interval.

It is well known that miRNAs regulate gene expression via inhibiting translation or degrading target mRNAs [38]. To better understand the biological functions of the differentially expressed miRNAs in the bleomycin-induced DNA damage repair of rice, the psRNATarget was used to predict potential miRNA target genes using the most abundant mature miRNA as queries. Using the strict standard described in the methodology, a total of 8731 target genes for the 150 miRNAs were predicted. Among them, 5666 and 317 target genes were predicted for 66 known and three novel up-regulated miRNAs, respectively (Table S4a), and 5933 and 524 target genes were predicted for 74 known and seven novel down-regulated miRNAs, respectively (Table S4b). As shown in Figure 6, 61 out of these differentially expressed miRNAs targeted 91 to 110 genes each; nine of these differentially expressed miRNAs had 21 to 40 target genes each; and 35 of these differentially expressed miRNAs regulated 71 to 90 target genes. These results suggest that miRNAs play a wide regulatory function in rice DDR.

**Figure 7:**
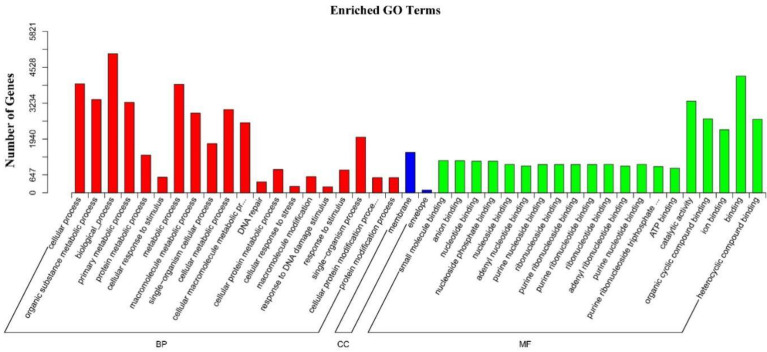
GO analysis for target genes of differentially expressed miRNAs. The horizontal axis depicts the three major categories including BP (biological process), CC (cellular component), and MF (molecular function), and 42 subcategories. The vertical axis depicts the actual number of the categorized target genes.

Gene ontology (GO) is an important bioinformatics analysis method used for determining various attributes of genes and gene products. GO annotations are divided into three major categories: biological process (BP), cellular component (CC), and molecular function (MF), which explain the biological role of proteins. Based on the distribution of target genes, the differentially expressed miRNAs were classified into 42 functional subcategory annotations, providing an overview of the ontology content. As shown in Figure 7, 20 of these subcategories were classified into ‘molecular function,’ 20 into ‘biological process,’ and 2 into ‘cellular component,’ respectively. As metabolic processes are important in responses to DNA damage for rice, it is understandable that metabolic processes and biological processes were the top two GO terms within BP. Notably, DNA repair and response to DNA damage stimulus subcategories are enriched, indicating that miRNAs may participate in the DDR and DNA repair process in rice. Regarding molecular function, the binding term and other binding term occupied the largest numbers of target genes. These results suggest that miRNAs play key roles in the regulation of transcription and translation processes, which is consistent with a report that miRNAs respond to DNA damage through gene transcription or post-translational regulation [39].

**Figure 8:**
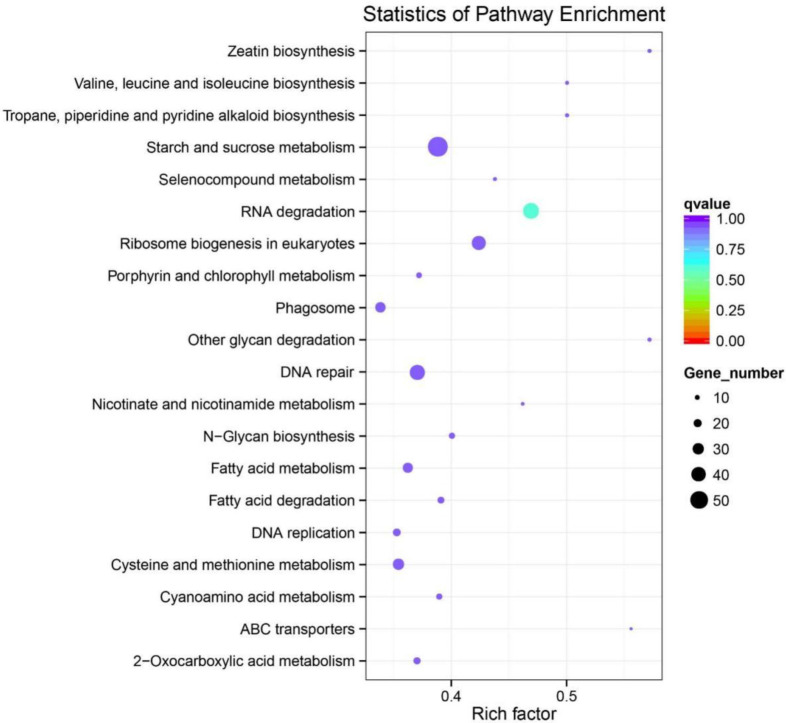
KEGG analysis of the top 20 enriched pathways. The coloring of the q-values indicates the reliability of the rich factor. The dot indicates the target genes involved in the pathway, and the size represents the number of genes where a larger dot represents a greater number of genes. The ordinate lists names of enrichment pathways. The abscissa indicates the rich factor.

The KEGG (Kyoto Encyclopedia of Genes and Genomes) library is a knowledge base of a systematic analysis of gene function, contact genomic information, and functional information [40]. The KEGG database can be used to predict the biological function of the differentially expressed miRNA target genes. In this study, the statistical enrichment of the gene candidates in KEGG pathways showed that the majority of target gene clusters were included in RNA degradation, other glycan degradation, starch and sucrose metabolism, nucleotide excision repair, cysteine and methionine metabolism, fatty acid metabolism, N-Glycan biosynthesis, base excision repair, and mismatch repair. (Table S5). From the top 20 enriched pathway terms, we can find that the main enriched factors included starch and sucrose metabolism, DNA repair, RNA degradation, and ribosome biogenesis in eukaryotes (Figure 8). It is worth noting that “DNA repair” was the most significantly enriched term with respect to gene number, indicating that miRNAs may be produced to respond to DNA damage by regulating the expression of target genes related to DNA repair in rice.

### 2.7. Validation of the miRNAs and Their Target Genes Involved in DNA Damage Repair in Rice

**Figure 9:**
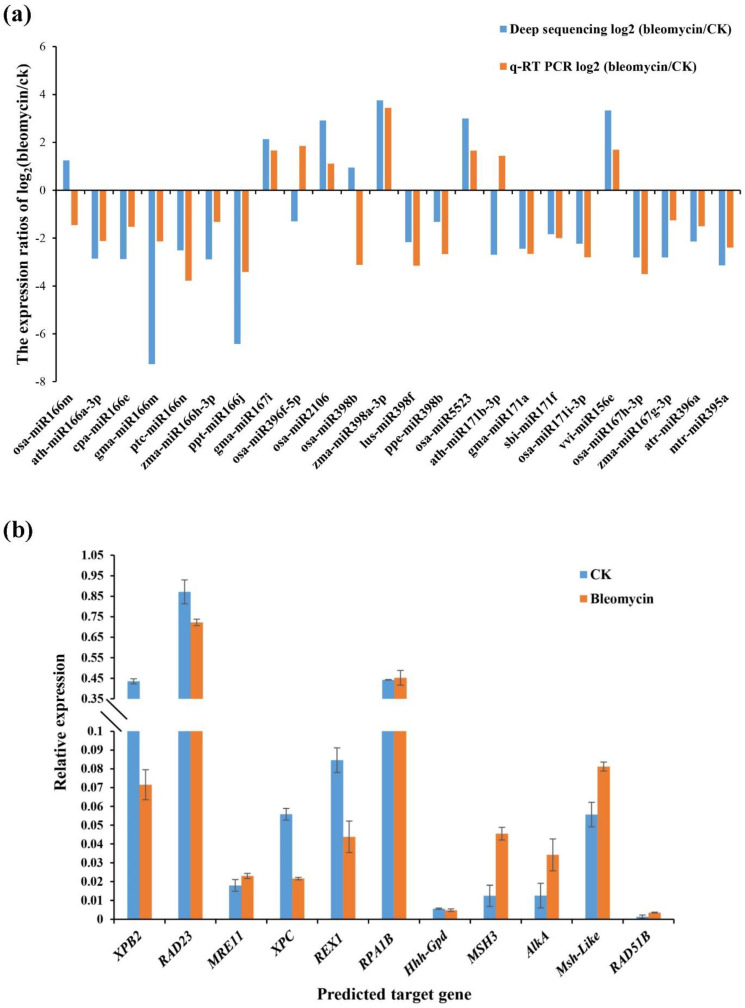
RT-qPCR validation of the expression levels of miRNAs and their gene targets. (**a**) The expression ratios [log2 (bleomycin/ck)] of the differentially expressed miRNAs along with target genes relating to DSB repair processes in rice were detected by RT-qPCR and deep sequencing; (**b**) Quantitative expression analyses of eleven target genes of selected miRNAs. The ubiquitin gene was used as the internal control for target gene expression. Each bar indicates the mean ± SE of triplicate assays.

To identify miRNAs responding to DNA damage repair in rice, 150 differentially expressed miRNAs were screened according to the KEGG database results. A total of 58 differential miRNAs were obtained, which targeted 41 potential target genes related to DNA repair (Table 4). Compared with the control group, thirty miRNAs were down-regulated in the bleomycin-treated rice roots, but 28 miRNAs were up-regulated as shown in Table 4. Based on the results, we selected 24 miRNAs with down- or up-regulated expression levels and 11 corresponding target genes for validating the expression patterns derived from the sequencing data using real-time quantitative PCR. As shown in Figure 9a, the relative transcript levels of 20 miRNAs, including ath-miR166a-3p, ptc-miR166n, gma-miR167i, lus-miR398f, osa-miR171i-3p, osa-miR5523, gma-miR171a, vvi-miR156e, and zma-miR167g-3p, were similar to those in the sequencing results, whereas four miRNAs showed expression patterns that contrasted with the sequencing results, including osa-miR166m, osa-miR396f-5p, osa-miR398b, and ath-miR171b-3p. Overall, the results of RT-qPCR for most miRNAs were consistent with the high-throughput sequencing.

Usually, miRNAs and their target genes have contrasting expression patterns. Consequently, we authenticated the expression levels of the target gene XPB2 for six miRNAs (osa-miR166m, ath-miR166a-3p, cpa-miR166e, gma-miR166m, ptc-miR166n, and zma-miR166h-3p), the gene RPA1B for four miRNAs (osa-miR398b, zma-miR398a-3p, lus-miR398f, and ppe-miR398b), the AlkA gene for three miRNAs (ath-miR171b-3p, gma-miR171a, and sbi-miR171f), the MSH3 gene for two miRNAs (osa-miR171i-3p and vvi-miR156e), the MSH-like gene for two miRNAs (osa-miR167h-3p and zma-miR167g-3p), the REX1 gene for two miRNAs (osa-miR396f-5p and osa-miR2106), and one potential target gene each (RAD23, XPC, Hhh-GPD, MRE11, RAD51B) for ppt-miR166j, gma-miR167i, osa-miR5523, atr-miR396a, and mtr-miR395a, respectively. As shown in Figure 9b, the expected opposing expression relationships were observed between miRNAs and their target genes, including the five miRNAs ath-miR166a-3p, cpa-miR166e, gma-miR166m, ptc-miR166n, and zma-miR166h-3p and their target gene XPB2, ppt-miR166j and its target gene RAD23, atr-miR396a and its target gene MRE11, gma-miR167i and its target gene XPC, osa-miR2106 and its target gene REX1, the three miRNAs ath-miR171b-3p, gma-miR171a, and sbi-miR171f and their target gene AlkA, osa-miR171i-3p and its target gene MSH3, the two miRNAs osa-miR167h-3p and zma-miR167g-3p and their target gene MSH-like, and mtr-miR395a and its target gene RAD51B. These results indicate that these miRNAs may participate in the process of DNA repair by regulating the expression of these predicted genes related to DNA repair. However, the expression of osa-miR166m and its target gene XPB2, osa-miR396f-5p and its target gene REX1, the four miRNAs osa-miR398b, zma-miR398a-3p, lus-miR398f, and ppe-miR398b and their target gene RPA1B, osa-miR5523 and its target gene Hhh-GPD, and vvi-miR156e and its target gene MSH3 show no contrasting expression patterns, indicating that these may not be the actual target genes of these miRNAs. This might be due to false prediction results from the highly repetitive sequences that match up to miRNAs in the gene database. Therefore, more experimental validation is needed to clarify the regulatory mechanism of miRNAs and their targets in the process of DNA repair in rice.

## 3. Discussion

Increasing evidence has indicated that miRNAs play important roles in DDR [39,41]. Nevertheless, little is known about the mechanisms of the small non-coding RNAs involved in this process in rice. In this study, we generated sRNA libraries of bleomycin-treated and the non-treated rice seedling roots, generating approximately 15 million clean reads per library. This indicates that we have generated a relatively complete small RNA library, which is convenient for identifying miRNAs, especially those specifically expressed in root cells that have undergone DNA damage. Of these sRNA sequences, more than 0.4 and 1.2 million unique sequences were obtained for the bleomycin treatment and control libraries, respectively. Notably, unannotated unique sRNAs accounted for a decent proportion of reads in the bleomycin treatment library, indicating that many potential miRNAs have not yet been functionally studied regarding their response to DNA damage in rice.

The vast majority of plant small RNAs are 21 or 24 nt in length. Every kind of plant has its own specific length distribution patterns of small RNAs. For example, many angiosperms have a major peak at 24 nt, including maize [42], potato [43], tomato [44], and Arabidopsis [45], while the length distribution patterns of small RNAs from gymnosperms, including Tasus chinensis, Pinus contorta, and seven other conifer species, show a major peak at 21 nt, with no obvious peak at 24 nt [46,47,48]. In the present study, the small RNA length distribution for the CK library showed a major peak at 24 nt which is consistent with the previous studies, and different from the bleomycin treatment library with the major peak at 19 nt. This suggests that small RNA length distribution patterns are highly conserved in the same plant species but differ considerably after suffering from abiotic stress. 

Previous studies have reported that a single miRNA can target multiple genes, or many miRNAs can regulate a single gene, suggesting the functional divergence of these miRNAs [10]. On average, every miRNA has 7, 19, and 17 target genes in Cavendish bananas, Ginkgo biloba, and Japanese apricot, respectively [2,10,49]. In our study, 40 percent of miRNAs targeted 91 to 110 genes and most miRNAs had more than 40 target genes. These differences might be due to a stronger regulatory ability of the miRNAs to respond to DNA damage in rice. Additionally, two differentially expressed miRNAs without a predicted gen target were identified, making it difficult to understand the function of these miRNAs. This also illustrates the limitation of the current mapping approach. It will be important to confirm the accurate function of these miRNAs through further experiments.

More and more evidence indicates that miRNAs might act as regulatory factors involved in the DDR processes [50,51,52,53,54,55,56,57,58]. In human cells, a large number of miRNAs have been demonstrated to play modulator roles in the regulation of DDR. For example, miR-18a and miR-412 can negatively regulate ATM expression and reduce the number of tumor cells to repair DNA damage after radiotherapy [53,55]; miR-25 and miR-30d have been shown to interact with p53, the master regulator of DDR, and its down-regulation leads to the reduction of apoptosis via suppressing expression of its target genes (p21, BAX, Puma) [52]. Furthermore, several studies have shown that DNA repair pathways are also influenced by miRNAs in human cancer cell lines [51,54,56]. Nevertheless, few studies have reported the potential roles of miRNAs in the regulation of DDR-associated genes in plants. XPB2, a component of the NER pathway, was shown to be the target of tae-miR1122c-3p in a transcriptome analysis of wheat anther [58]. Another component of the NER pathway gene, namely RAD23, was predicted to be the target of miR477 in switchgrass [50]. The MRE11 gene, belonging to the MRN complex, which plays a central role in DSB repair was identified as the target gene of miR5261 in Mg-deprived roots [57]. In this study, by further identifying differentially expressed miRNAs and their corresponding target genes, we identified some promising candidates potentially involved in the process of DNA damage repair. It is interesting that XPB2, RAD23, and MRE11 are also identified as target genes of differentially expressed miRNAs, but the corresponding miRNAs are quite distinct from the previous report [39]. XPB2 was predicted to be targeted by the miR166 miRNA family (osa-miR166m, ath-miR166a-3p, cpa-miR166e, gma-miR166m, ptc-miR166n, and zma-miR166h-3p). RAD23 and MRE11 were predicted to be targeted by ppt-miR166j and atr-miR396a, respectively. A further RT-qPCR analysis revealed that the expression of the miR166 family had opposite expression patterns, except for osa-miR166m and target gene XPB2, ppt-miR166j and target gene RAD23, and atr-miR396a and target gene MRE11. These results indicate that the same gene related to DNA damage repair may be regulated by different miRNAs among various plant species. In addition, there are other genes worth noting, including the following: XPC, the predicted target gene of gma-miR167i, is thought to be one of the first proteins to recognize DNA damage during global genomic repair (GGR) which is a sub-pathway of NER [59]; MSH3, the predicted target gene of osa-miR171i-3p is required to remove nonhomologous DNA ends during both the initiation of gene conversion and the resolution of SSA intermediates that are initiated by DSBs [60]; and RAD51B, the predicted target gene of mtr-miR395a, is involved in the repair of double-strand DNA breaks via homologous recombination in Arabidopsis [61]. Compared with the expression level of predicted target genes XPC, MSH3, and RAD51B, the expression levels of the corresponding miRNAs: gma-miR167i, osa-miR171i-3p, and mtr-miR395a show contrasting expression patterns, according to the deep sequencing and RT-qPCR results. These results indicate that gma-miR167i, osa-miR171i-3p, and mtr-miR395a may play important roles in the DNA repair process in rice.

Previous studies have shown that DNA damage frequently triggers death by apoptosis, of which miRNAs also play important regulatory roles [62]. P53, a tumor suppressor, is targeted by miR375 and can promote cell apoptosis. In humans, miR375 can inhibit the expression of P53, thereby preventing the apoptosis of motor neurons when DNA damage occurs [63]. ING5 is the target gene of miR1307, and miR1307 can inhibit apoptosis in ovarian cancer cells by down-regulating the expression of ING5 [64]. There are more in-depth studies in animal cell systems, but few studies in plants. Our research found hbr-miR6173, which regulates the target gene of E2 ubiquitin ligase (LOC_Os03g57790), is significantly up-regulated after bleomycin treatment. Expression of the E2 protein causes both cell cycle arrest and apoptosis in HeLa cells [65]. Furthermore, RT-qPCR indicated inverse expression patterns between miR6173 and its target gene E2 in samples from both the control and bleomycin treatment (Figure S2). Therefore, miR6173 may protect cells from apoptosis through the translational repression of the E2 gene related to apoptosis in rice. Further experiments involving the experimental knockout of these miRNAs and their gene targets are needed to determine their functions in DNA damage repair in rice.

## 4. Materials and Methods

### 4.1. Plant Materials and Sample Collection

The seeds used in this study were from the japonica rice cultivar ‘Yandao 8’. All plants were grown in a paddy field under normal growth conditions at Yangzhou University, China. Sterilized seeds were cultured on 1/2 MS solid medium, at 28 °C under a 13 h light/11 h dark photoperiod. Twelve-day-old seedlings were transferred to water with 0 or 10 μg/mL bleomycin (Yuanye Biology, Shanghai, China) for 24 h [66]. Roots of these seedlings were harvested and stored at −80 °C for further use.

### 4.2. Measurement of Effects of Bleomycin on Rice Growth

Sterilized seeds were germinated on 1/2 MS solid medium contained 0 or 10 μg/mL bleomycin. After culture in an incubator with a 13 h light/11 h dark photoperiod for 12 days, root and shoot lengths of the seedlings were measured.

### 4.3. Immunization

After exposing rooted seedlings to 10 μg/mL bleomycin for 3 h (untreated seedlings served as the control), the roots (2 cm) were collected and fixed using 4% paraformaldehyde (W/V) for 30 to 50 min. After washing twice with 1 × PBS solution, the root tips were crushed on a slide then frozen with liquid nitrogen for 30 s, after which the cover glass was removed and the slide incubated at 37 °C for 5 h with 50 μL of 1 × TNB buffer (0.1 M Tris-HCl, pH 7.5, 0.15 M NaCl, 0.5% blocking regent) containing 0.25 μL γH2AX antibody [67]. The slide was then washed in 1×PBS three times, 5 min each, on a shaker. After drying, 50 μL of 1 × TNB buffer containing 0.25 μL of Texas-real conjugated rabbit antibody (Invitrogen, Camarillo, CA, USA was added then incubated for 1 h in a warm bath (37 °C) in the dark, and finally rinsed three times with 1×PBS. After drying for 5 min, 10 μL of DAPI was used to stain each slide. Images were observed and captured with a fluorescence microscopy (Olympus DP80). Interphase nuclei were selected for pictures and quantitative analyses.

### 4.4. RNA Extraction and Deep Sequencing

Total RNA was extracted from frozen root samples using the RNAsimple Total RNA kit (TIANGEN, DP419, Beijing, China) according to the manufacturer’s instructions. Nine libraries were constructed using the Illumina TruSeq Small RNA kit [68]. The Illumina high-throughput sequencing platform was used to sequence the enriched 18-32 nt small RNA fragments. The protocol is as follows: 1 μg of eukaryotic total RNA was used as the starting material; ligation of the 3′ and 5′ end connectors (Truseq TM Small RNA sample prep Kit); random primers were used for 1st strand cDNA synthesis (Illumina, San Diego, CA, USA); library enrichment (PCR11-12 cycles); library purification (6% Novex TBE PAGE gel, 1.0 mm, 10 well); TBS380 (Picogreen) quantification, mixed on the machine according to the data ratio; bridge PCR amplification on bot to generate clusters; Illumina sequencing [69]. All reactions were performed in triplicate.

### 4.5. Bioinformatics Analysis of Sequencing Data

Quality control of the sequencing data was carried out to determine the amount and base quality of the original data. Adaptor, low quality tags and discarded sequences <18 nt were filtered out. Modified sequences from 18 nt to 30 nt from the resulting clean read data were used for further analyses. First, tRNAs, snoRNAs, scRNAs, snRNAs, and rRNAs were removed from the sRNA sequences by aligning to sequences in the Rfam (version 10.1) database. The remaining sRNA sequences were compared against rice ncRNAs deposited in the NCBI GenBank and Rfam10.0 databases. Previously known miRNAs in these sRNA sequences were identified by comparison with known plant miRNAs deposited in the miRBase 19.0. Lastly, sequences that did not map to known miRNAs were used to predict potentially novel miRNAs. Potentially novel miRNAs were identified using MIREAP software, followed by the prediction of secondary structures using RNAfold software. The criteria chosen for stem-loop hairpins were those described by Meyers et al. and Wang et al. [37,70].

### 4.6. Identifying Differentially Expressed miRNAs

The frequency of miRNAs in the two libraries were normalized using the following formula: transcripts per million (TPM) normalized expression = initial miRNA count/total count of clean reads × 1,000,000. Following normalization, miRNA expression profiles among the two sRNA libraries (control and bleomycin treatment) were compared. Normalized read counts of the two samples equal to zero were set to 0.01. Normalized read counts of the two samples that were less than 1 were excluded from the analysis of differential expression due to their expression being too low. Fold-changes between the bleomycin and control treatments were calculated as follows: fold-change = log2 (bleomycin treatment/control). The p-value was calculated using the formula below, where N1 and N2 represent the total counts of clean reads of a given miRNA in the two libraries (control and bleomycin treatment), respectively; x and y represent the normalized expression levels of a given miRNA in the two libraries, respectively.

$$p\left(x|y\right)={\left(\frac{N_{2}}{N_{1}}\right)}^{y}\frac{\left(x+y\right)!}{x!y!{\left(1+\frac{N_{2}}{N_{1}}\right)}^{\left(x+y+1\right)}}$$

$$C(y\leq y_{min}|x)=\overset{y\leq y_{min}}{\underset{y=0}{\sum }}p\left(y|x\right)$$

$$D(y\geq y_{max}|x)=\overset{\infty }{\underset{y\geq y_{max}}{\sum }}p\left(y|x\right)$$

### 4.7. Prediction of Target Genes of miRNAs

We used the web-based psRNA Target software (http://plantgrn.noble.org/psRNATarget/ accessed on 14 September 2021) and psRobot to identify putative targets for known and novel miRNAs. Sequences from the *Oryza sativa* transcriptome database were used as a custom target database. The criteria for target prediction of each miRNA were based on those suggested by Allen et al. and Schwab et al. [71,72].

### 4.8. RT-qPCR Confirmation of Differentially Expressed miRNAs and Their Possible Targets

Total RNA was extracted from previously frozen plant tissues using RNAsimple Total RNA kit. For each examined miRNA, 1 μg of total RNA was used as the template for reverse transcription using the miRcute Plus miRNA Fist-Strand cDNA kit (TIANGEN, Beijing, China); the U6 gene was used as the internal control. Reverse transcription was performed using the following conditions: 42 °C for 60 min, 95 °C for 3 min, hold at 4 °C. For target genes, 1 μg of total RNA was used for synthesis with HiScript II Q Select RT SuperMix for qPCR (Vazyme, Nanjing, China) and an oligo (dT) 23 primer, and the gene UBQ was used as an internal control. Reverse transcription was performed using the following conditions: 50 °C for 15 min, 85 °C for 5 s, hold at 4 °C. QPCR was performed on the Bio-Rad CFX96 Real-time PCR platform. The MiRcute miRNA qPCR Detection kit (TIANGEN, Beijing, China) and ChamQ SYBR qPCR Master Mix (Vazyme, Nanjing, China), respectively, were used for the RT-qPCR assays according to the manufacturer’s protocol. The relative expression of miRNA and its target genes was calculated by the 2^–∆∆Ct^ method. All primers used are listed in Supplementary Table S6.

## 5. Conclusions

To better understand the roles of miRNAs in rice DNA damage repair, we constructed two sRNA libraries of rice roots with or without bleomycin treatment. We identified a total of 513 known miRNAs and 72 novel miRNAs in two libraries using Illumina Hiseq2000 sequencing technology. A total of 140 known miRNAs and 10 novel differentially expressed candidate miRNAs were identified that may have important roles in response to bleomycin-induced DNA damage in rice. A total of 41 corresponding target genes were predicted as targets of these miRNAs. A GO enrichment analysis of these target genes indicated some were involved in DNA repair processes. We confirmed the sequencing results of selected differentially expressed miRNAs and their potential gene targets via RT-qPCR. While the exact functions of these differentially expressed miRNAs requires further investigation, the findings of this study provide valuable information for a further functional analysis of the miRNAs produced in response to DNA damage in rice.

## Figures and Tables

**Table 1:** Sequencing results of sRNAs derived from two sample libraries.

Read Types	Small RNA Libraries		Average
	Control Group	Bleomycin Treatment Group	
Raw reads	17,369,981 (100%)	14,716,232 (100%)	16,043,106
N% > 10% reads	1287 (0.01%)	220 (0.00%)	753.5
Low quality reads	0 (0.00%)	0 (0.00%)	0
Adaptor reads	256,677 (1.48%)	456,550 (3.1%)	356,613.5
with ployA/T/G/C reads	20,295 (0.12%)	11,446 (0.08%)	15,870.5
Clean reads	17,091,722 (98.39%)	14,248,017 (96.82%)	15,669,869

**Table 2:** Classification of small RNAs identified in rice roots.

Read Types	Small RNA Libraries		Average
	Control Group	Bleomycin Treatment Group	
**Total**	12,819,497.67 (100.00%)	4,736,578.33 (100.00%)	8,778,038
Known_miRNA	246,484 (1.93%)	28,315.33 (0.57%)	137,399.67
rRNA	4,822,711 (37.63%)	865,373.67 (19.69%)	2,844,042.33
tRNA	59 (0.00%)	14 (0.00%)	36.5
snRNA	11,310.33 (0.09%)	4470 (0.10%)	7890.17
snoRNA	79,324 (0.61%)	23,826.33 (0.54%)	51,575.17
Repeat	942,610.33 (7.33%)	191,491 (3.78%)	567,050.67
NAT	135,339.33 (1.06%)	307,870 (5.87%)	221,604.67
Novel_miRNA	1629 (0.01%)	284.33 (0.01%)	956.67
Exon:+	3,407,711.67 (26.61%)	1,194,342 (25.35%)	2,301,026.83
Exon:-	45,844 (0.36%)	17,892.33 (0.36%)	31,868.17
Intron:+	101,195.67 (0.79%)	376,482.67 (7.70%)	238,839.17
Intron:-	102,132.33 (0.80%)	91,365.33 (1.83%)	96,748.83
other	2,923,147 (22.77%)	1,634,851.33 (34.21%)	2,278,999.17

All sRNAs that aligned to the reference sequence; rRNA/tRNA/snoRNA/snRNA considered; NAT: natural antisense short interfering RNA; Exon: +/exon: −/intron: +/intron: −: the exon/intron of positive and negative chains considered.

**Table 3:** Information of known miRNA families and their transcript abundance identified in the two libraries.

Family	No. of Members	miRNA Reads		Normalized Reads		Fold Change
		CK	Bleomycin	CK	Bleomycin	Log2(Bleomycin/CK)
miR171	29	343	44	26.76	9.22	−1.54
miR156	27	1355	1598	105.67	337.44	1.68
miR166	24	159,766	7058	12,462.71	1490.18	−3.06
miR159	21	113,946	3148	8888.52	664.54	−3.74
miR169	20	324	74	25.27	15.62	−0.69
miR167	19	6488	653	506.08	137.93	−1.88
miR396	19	12,312	1553	960.41	327.80	−1.55
miR812	13	126	36	9.83	7.60	−0.37
miR397	12	62,874	1547	4904.56	326.54	−3.91
miR172	10	6	15	0.47	3.17	2.76
miR164	10	71	61	5.56	12.81	1.20
miR398	10	3104	1433	242.11	302.61	0.32
miR395	10	89	1	6.97	0.14	−5.64
miR160	9	312	366	24.31	77.20	1.67
miR319	9	1018	80	79.41	16.96	−2.23
miR408	8	36,263	3877	2828.76	818.59	−1.79
miR444	7	463	155	36.12	32.79	−0.14
miR1861	7	106	38	8.24	8.09	−0.03
miR162	6	5316	859	414.65	181.28	−1.19
miR393	6	7	1	0.57	0.14	−2.02
miR390	5	76	7	5.95	1.41	−2.08
miR1846	5	12	6	0.91	1.34	0.56
miR1863	5	23	12	1.77	2.53	0.52
miR535	4	46	12	3.56	2.53	−0.49
miR1432	4	364	202	28.37	42.65	0.59
miR2118	3	1	2	0.08	0.35	ND
miR810	3	33	28	2.60	5.98	1.20
miR482	3	39	0	3.04	0.01	−8.25
miR168	3	358	138	27.93	29.21	0.06
miR399	3	3	0	0.23	0.01	ND
miR2863	3	31	31	2.42	6.54	1.44
miR1862	3	4565	469	356.12	99.09	−1.85
miR1120	3	4	2	0.31	0.42	ND
miR529	3	4	0	0.31	0.01	ND
miR1882	2	3831	9339	298.84	1971.68	2.72
miR3522	2	2	0	0.16	0.01	ND
miR1878	2	8	7	0.62	1.48	1.24
miR1428	2	3	0	0.23	0.01	ND
miR2871	2	17	8	1.33	1.69	0.35
miR1437	2	6	1	0.47	0.14	−1.73
miR528	2	2047	699	159.68	147.57	−0.11
miR3630	2	101	98	7.88	20.69	1.39
miR1440	2	2	0	0.16	0.01	ND
miR2873	2	5	2	0.39	0.42	0.11
miR5143	1	14	3	1.09	0.56	−0.96
miR5160	1	0	1	0.03	0.21	ND
miR1122	1	1	1	0.05	0.14	ND
miR439	1	2	2	0.13	0.35	ND
miR2275	1	0	1	0.01	0.21	ND
miR2121	1	2	1	0.16	0.14	ND
miR814	1	4	7	0.29	1.55	2.44
miR5539	1	1	0	0.08	0.01	ND
miR820	1	1066	197	83.15	41.59	−1.00
miR827	1	2	0	0.18	0.07	ND
miR1511	1	10	0	0.78	0.01	−6.29
miR5157	1	1	0	0.08	0.01	ND
miR1883	1	185	2	14.46	0.49	−4.88
miR1319	1	6	1	0.44	0.21	−1.07
miR5534	1	1	0	0.08	0.01	ND
miR8155	1	10	0	0.78	0.01	−6.29
miR5148	1	4	1	0.31	0.21	ND
miR5079	1	30	71	2.34	15.06	2.69
miR394	1	35	3	2.73	0.56	−2.28
miR9863	1	1	0	0.08	0.01	ND
miR530	1	1	1	0.08	0.14	ND

Normalized reads formula: normalized read count = (miRNA read count/total read count) × 1,000,000. The total read count in the CK and bleomycin treatment library are 12,819,498 and 4,736,578, respectively (Table 2). For further analysis, the normalized read count with a value of 0 was set to 0.01. If the read count of an miRNA was less than five in both libraries, differential expression analysis was not performed due to the expression level being too low, which is indicated as ND.

**Table 4:** List of differentially expressed miRNAs and target genes relating to DNA repair identified by KEGG pathway analysis.

miRNA	Differently Expressed	Target Gene	Protein Feature
osa-miR319a-3p.2-3p	Down	LOC_Os04g54500	POLGAMMA2, putative, expressed
osa-miR5513	Down	LOC_Os12g07720	RFC4-Putative clamp loader of PCNA, replication factor C subunit 4, expressed
osa-miR166m	Up	LOC_Os01g49680	DNA repair helicase XPB2, putative, expressed2
ath-miR166a-3p	Down		
cpa-miR166e	Down		
gma-miR166m	Down		
ptc-miR166n	Down		
zma-miR166h-3p	Down		
osa-miR5149	Down	LOC_Os03g03650	POLD2-Putative DNA polymerase delta complex subunit, expressed
ptc-miR6478	Up	LOC_Os11g08330	POLD1-Putative DNA polymerase delta catalytic subunit, expressed
ppt-miR166j	Down	LOC_Os06g15360	RAD23 DNA repair protein, putative, expressed
osa-miR1872	Down	LOC_Os01g01700	Ring- finger domain protein; RING-box protein 1
novel_11	Down	LOC_Os08g07840	PolI-like DNA polymerase, putative, expressed
osa-miR5807	Down	LOC_Os02g53500	RFC5-Putative clamp loader of PCNA, replication factor C subunit 5, expressed
gma-miR167i	Up	LOC_Os08g33082	DNA-repair protein complementing XP-C cells, putative, expressed
zma-miR171b-3p	Down	LOC_Os11g36390	RFC1-Putative clamp loader of PCNA, replication factor C subunit 1, expressed
aau-miR160	Down	LOC_Os02g53680	Replication Protein A
zma-miR164h-5p	Up	LOC_Os02g30800	POLE1-Putative DNA polymerase epsilon catalytic subunit, expressed
osa-miR396f-5p	Down	LOC_Os05g10980	REX1 DNA Repair family protein, expressed
osa-miR2106	Up		
osa-miR398b	Up	LOC_Os03g11540	RPA1B-Putative single-stranded DNA binding complex subunit 1, expressed
zma-miR398a-3p	Up		
lus-miR398f	Down		
ppe-miR398b	Down		
zma-miR164h-5p	Up	LOC_Os03g01100	endonuclease, putative, expressed
osa-miR3979-5p	Up	LOC_Os01g49180	ATP dependent DNA ligase family protein, putative, expressed
osa-miR5523	Up	LOC_Os03g57870	clamp loader of PCNA; replication factor C subunit 3
mtr-miR171c	Up	LOC_Os02g47605	expressed protein
han-miR3630-3p	Up	LOC_Os03g63870	expressed protein
ath-miR167d	Down	LOC_Os04g42990	suppressor of stem-loop protein 1, putative, expressed
bna-miR167d	Down		
osa-miR167d-5p	Down		
ath-miR167a-5p	Up		
osa-miR5523	Up		
ptc-miR167f-5p	Up		
vvi-miR167c	Up		
aqc-miR171f	Down	LOC_Os08g07850	PolI-like DNA polymerase, putative, expressed
osa-miR166a-5p	Down	LOC_Os03g52750	rice cyclin gene
osa-miR166d-5p	Down		
osa-miR3979-5p	Up	LOC_Os10g42400	expressed protein
osa-miR3979-5p	Up	LOC_Os03g10780	flap endonuclease, putative, expressed
hvu-miR397a	Down	LOC_Os08g21330	formamidopyrimidine-DNA glycosylase, putative, expressed
ath-miR156a-5p	Up		
cca-miR156b	Up		
nta-miR156f	Up		
novel_199	Up		
osa-miR1872	Down	LOC_Os11g16580	endonuclease III-like protein 1, putative, expressed
osa-miR5523	Up	LOC_Os12g10850	hhH-GPD superfamily base excision DNA repair protein, putative, expressed
mtr-miR395g	Down	LOC_Os02g53430	DNA-3-methyladenine glycosylase, putative, expressed
ptc-miR6478	Up	LOC_Os09g36530	endonuclease/exonuclease/phosphatase family protein, putative, expressed
ath-miR171b-3p	Down	LOC_Os05g49250	hhH-GPD superfamily base excision DNA repair protein, putative, expressed
gma-miR171a	Down		
sbi-miR171f	Down		
osa-miR171i-3p	Down	LOC_Os04g58630	DNA mismatch repair protein MSH3, putative, expressed
vvi-miR156e	Up		
osa-miR167h-3p	Down	LOC_Os05g19270	MSH-like DNA mismatch repair protein, putative, expressed
zma-miR167g-3p	Down		
nta-miR156f	Up	LOC_Os09g37930	expressed protein
pab-miR1863	Up	LOC_Os09g32450	DNA topoisomerase 3 protein, putative, expressed
atr-miR396a	Up	LOC_Os04g54340	double-strand break repair protein MRE11, putative, expressed
osa-miR2880	Down	LOC_Os01g21440	expressed protein
osa-miR5484	Up		
osa-miR5079a	Up	LOC_Os08g08030	double-strand break repair protein MRE11, putative, expressed
ppe-miR396a	Up	LOC_Os03g43850	recA protein, putative, expressed
mtr-miR395a	Down	LOC_Os05g03050	DNA repair protein Rad51, putative, expressed
osa-miR396g	Up	LOC_Os01g71960	endonuclease, putative, expressed

## Data Availability

All of the raw data of small RNA sequences in this research have been deposited in the Sequence Read Archive (SRA) database of NCBI (SRR15881140-SRR15881145).

## Supplementary Materials

The following are available online at https://www.mdpi.com/article/10.3390/ijms22189958/s1.
