# Supplementary material for: Deep Sequencing Discovery and Profiling of Known and Novel miRNAs Produced in Response to DNA Damage in Rice
Source: Int J Mol Sci. 2021 Sep 15;22(18):9958. doi: 10.3390/ijms22189958 (PMC8472271; doi:10.3390/ijms22189958)

Figure S1. The predicted hairpin structures of 72 novel miRNAs.

novel_11

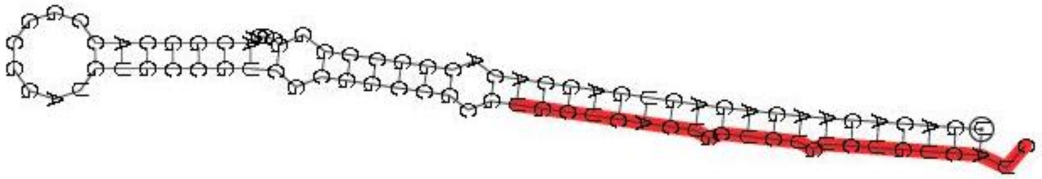

novel_14

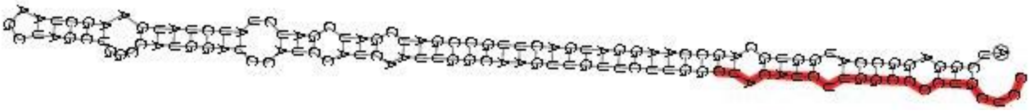

novel_29

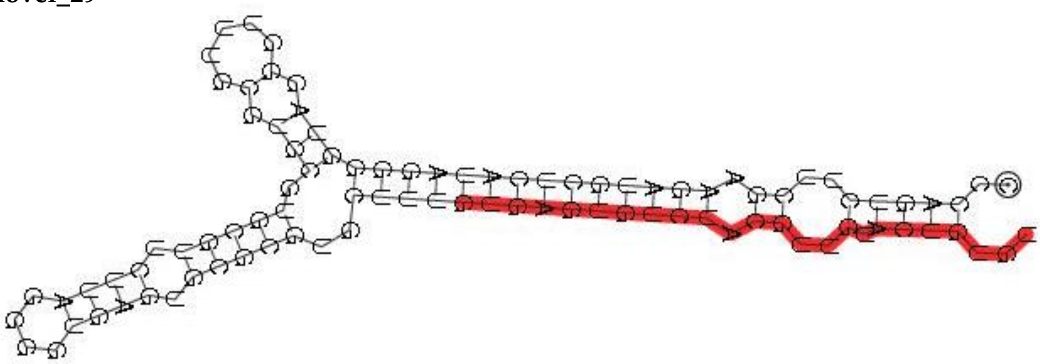

novel_37

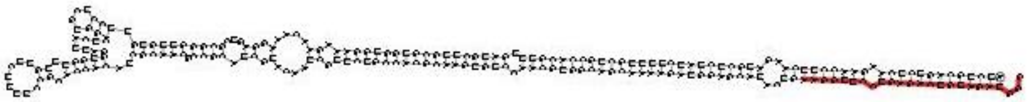

novel_40

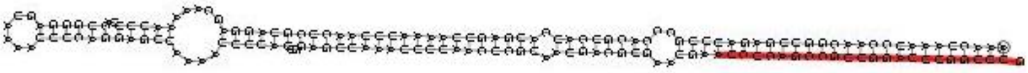

novel_51

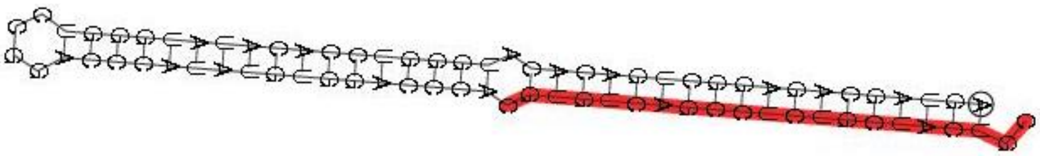

novel_52

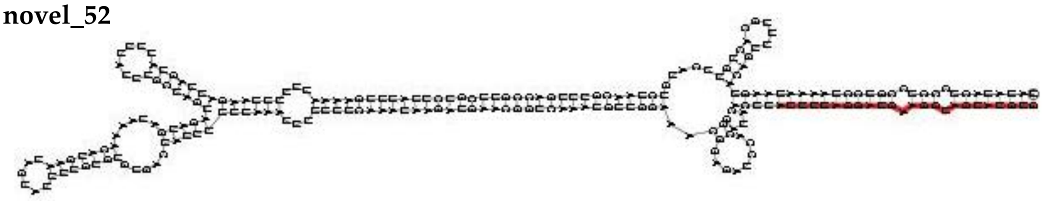

novel_56

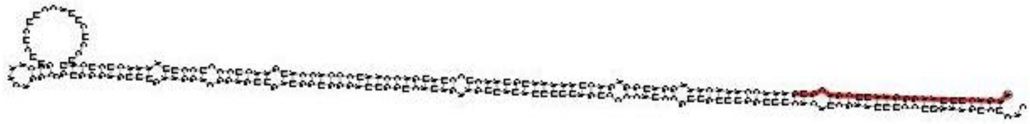

novel_58

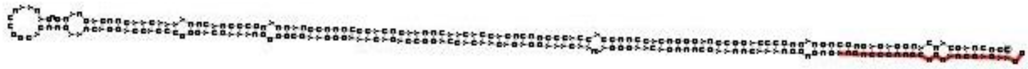

novel_68

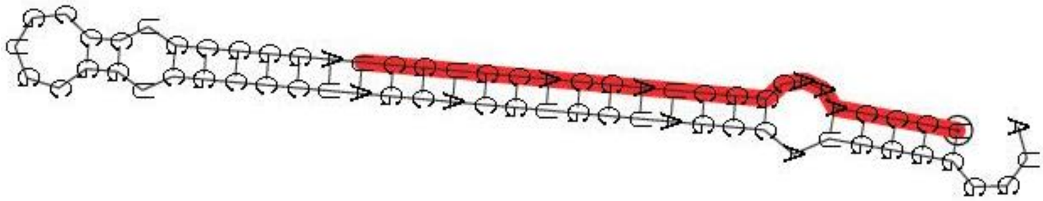

novel_72

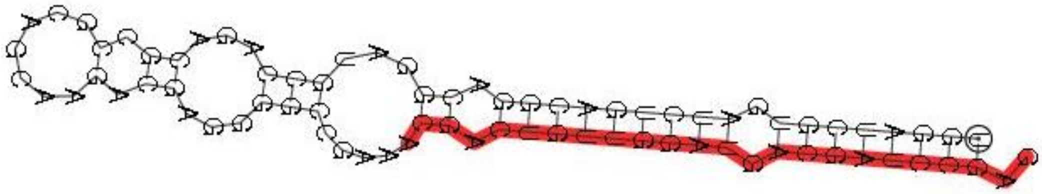

novel_76

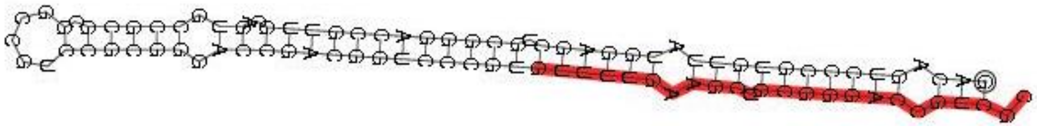

novel_78

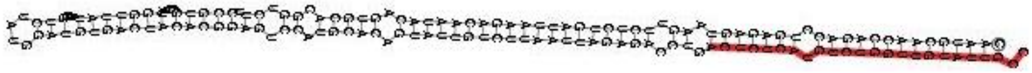

novel_79

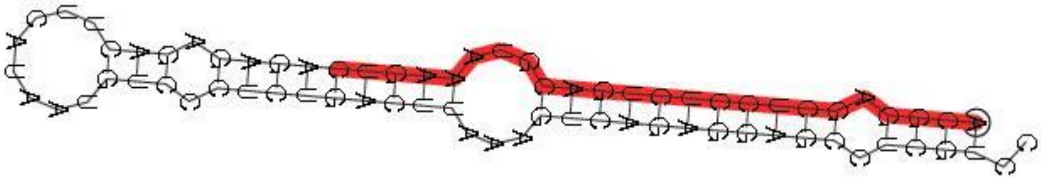

novel_83

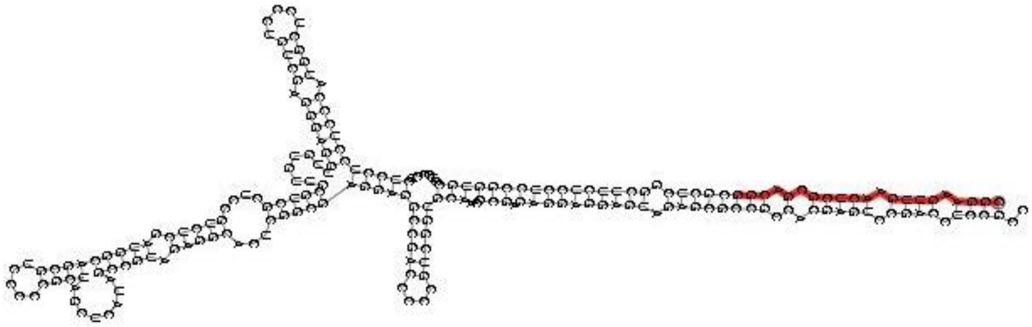

novel_87

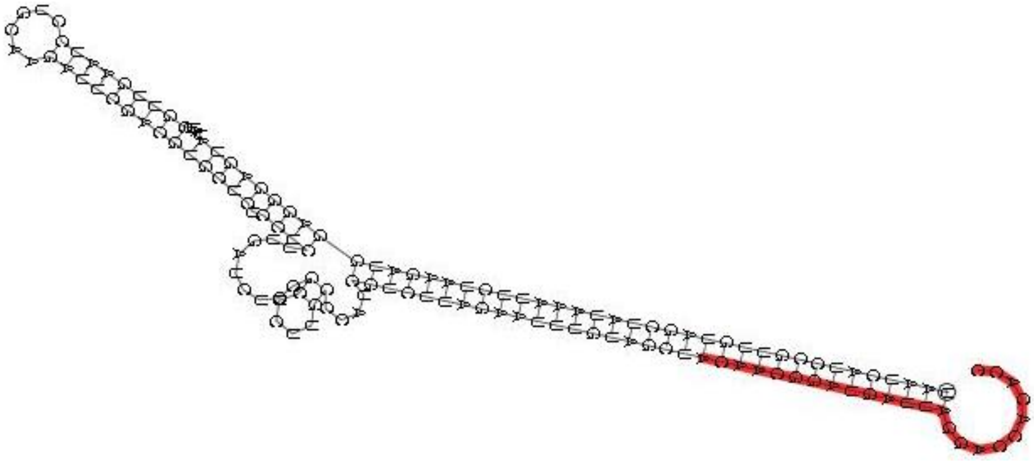

novel_93

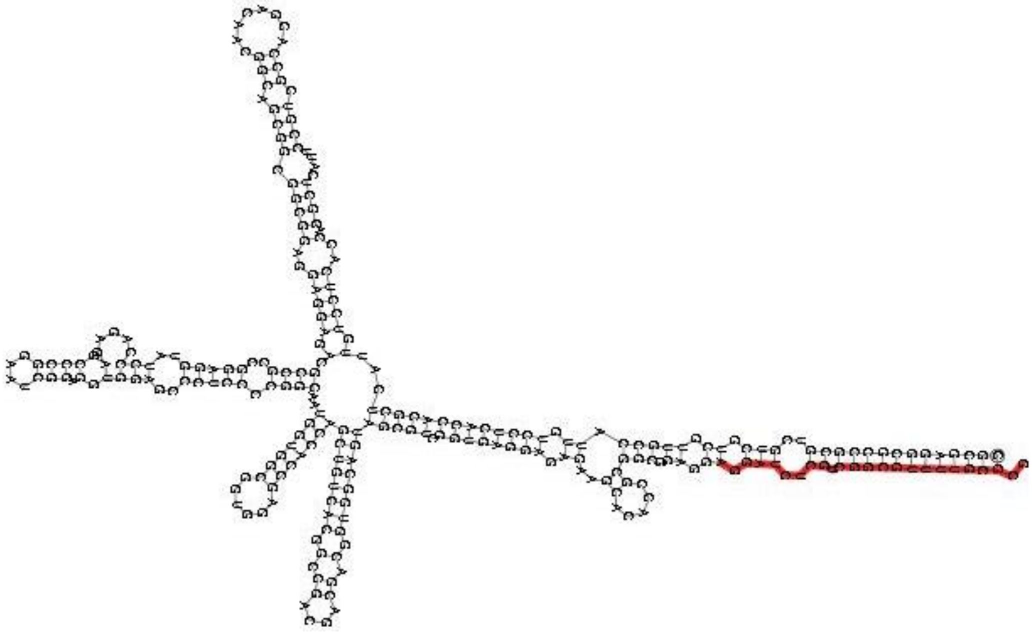

novel_95

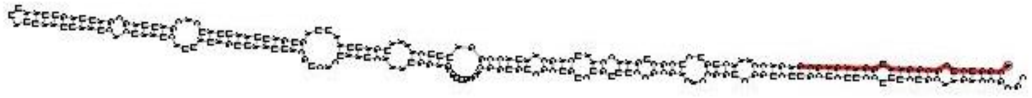

novel_97

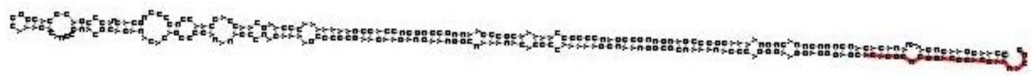

novel_98

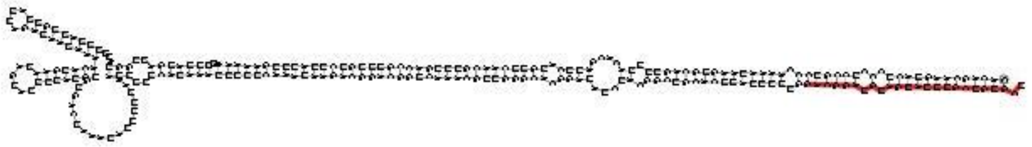

novel_100

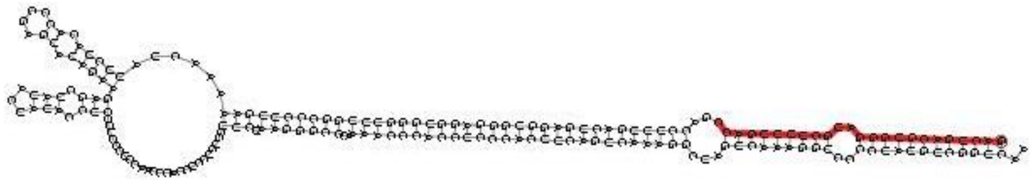

novel_105

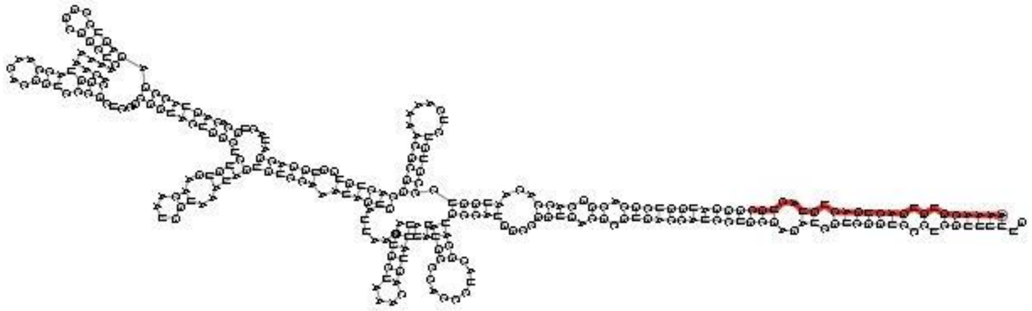

novel_107

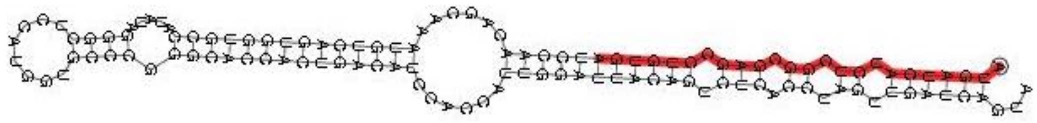

novel_110

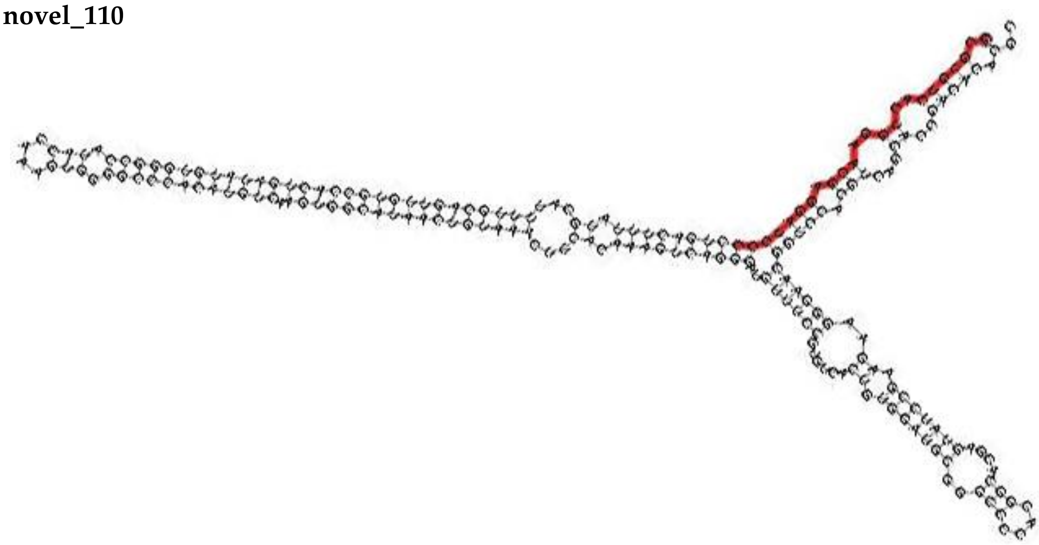

novel_112

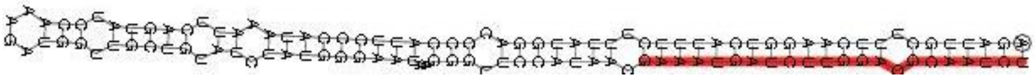

novel_113

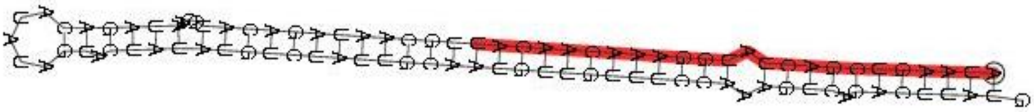

novel_116

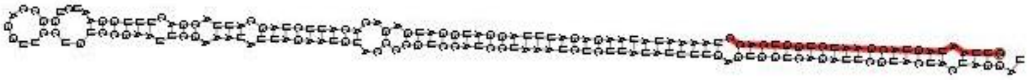

novel_119

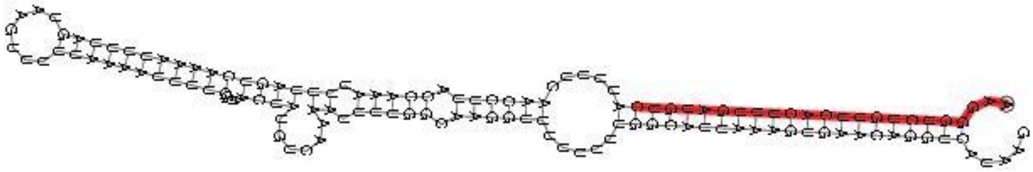

novel_121

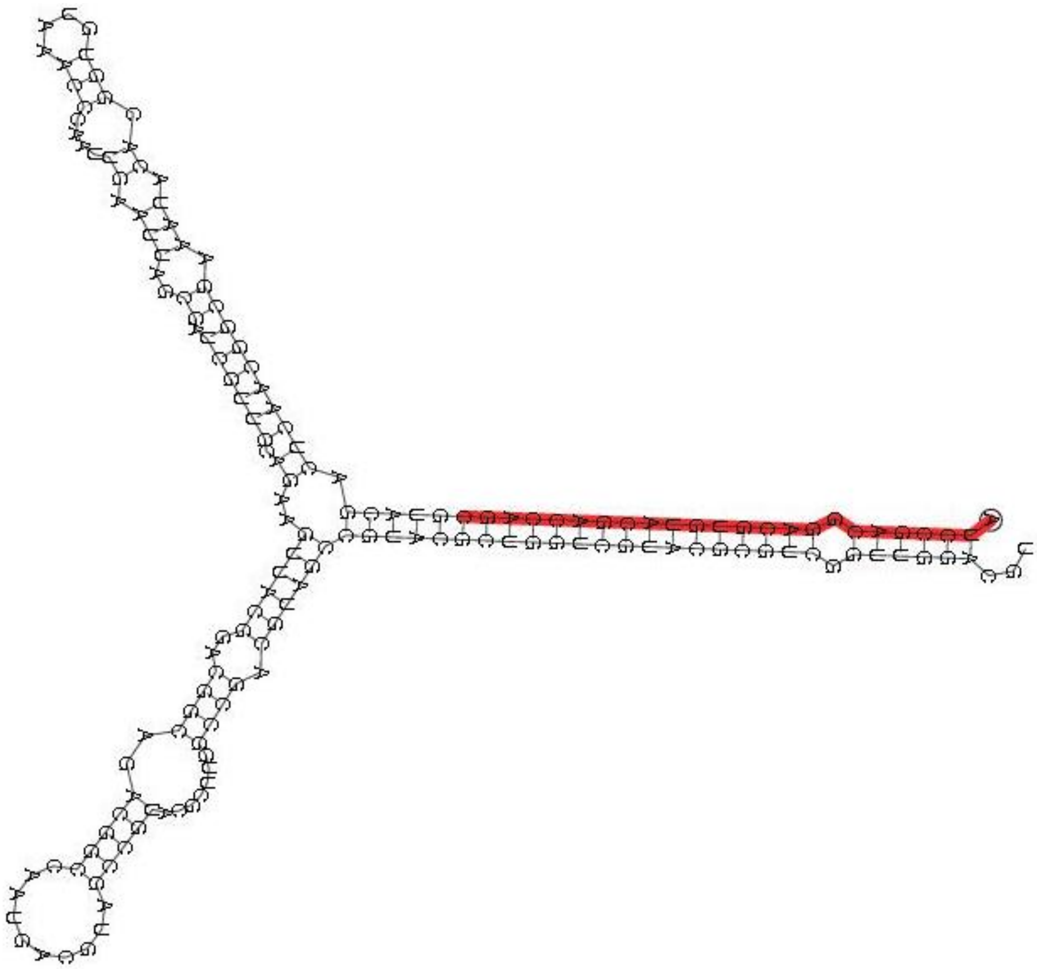

novel_122

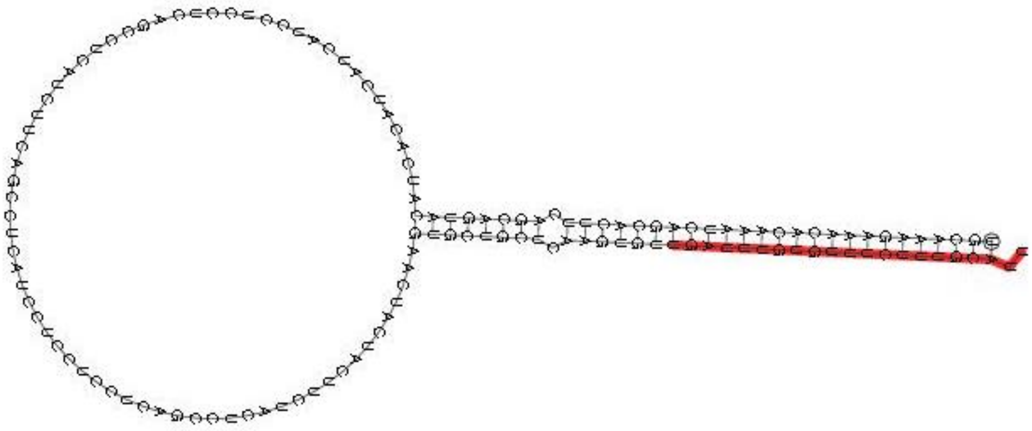

novel_131

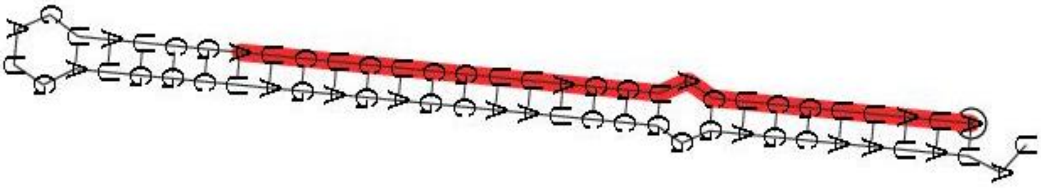

novel_132

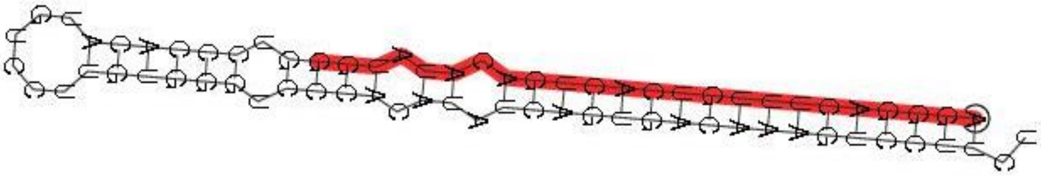

novel_133

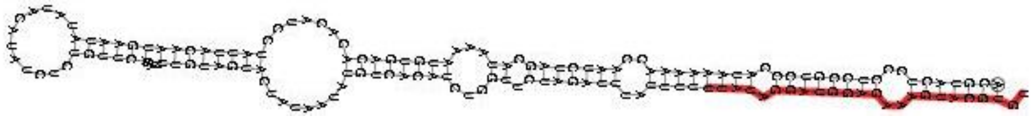

novel_139

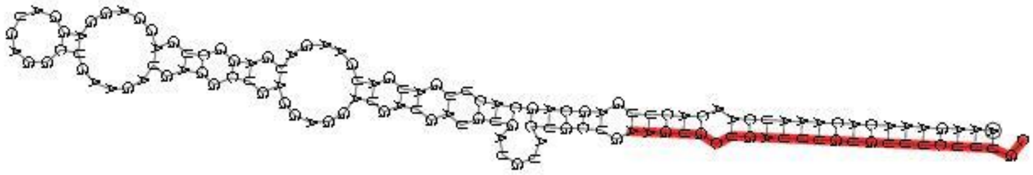

novel_146

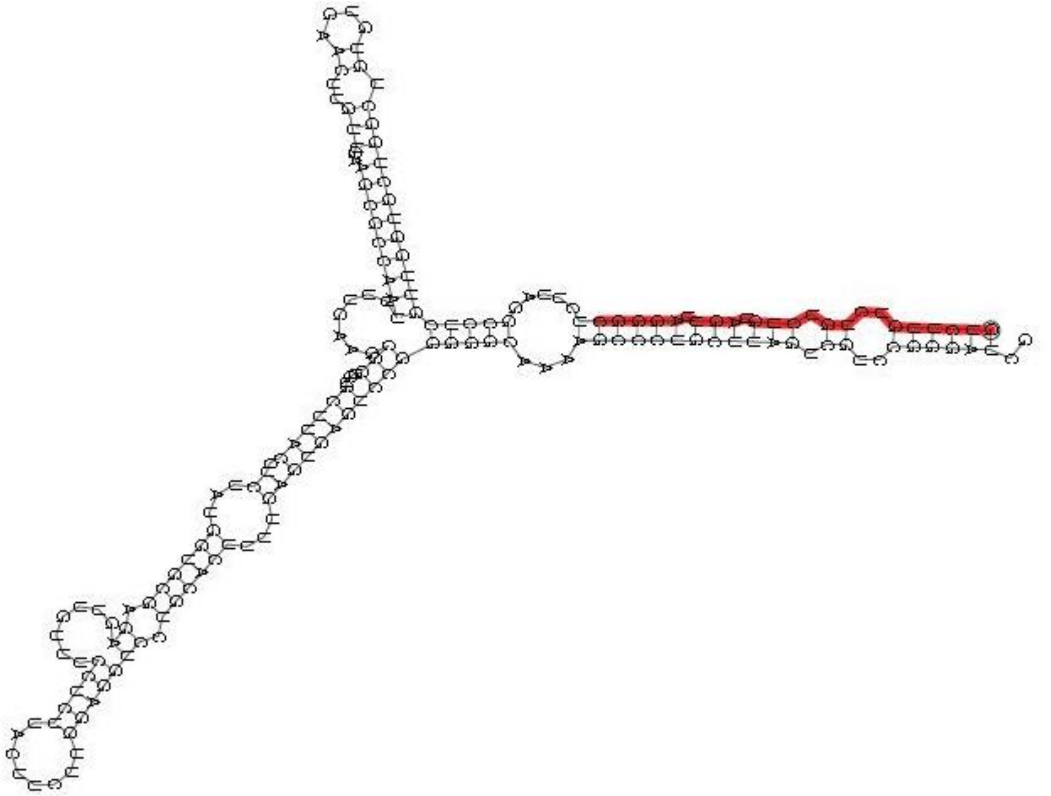

novel_149

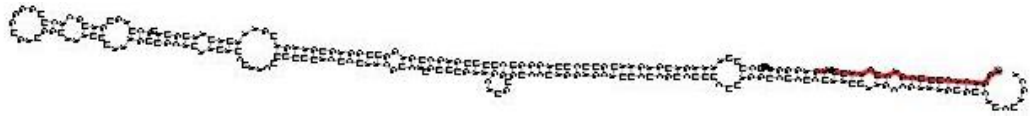

novel_150

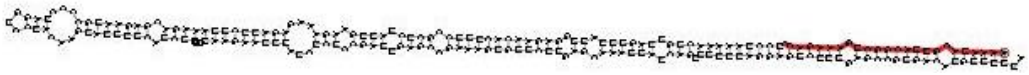

novel_154

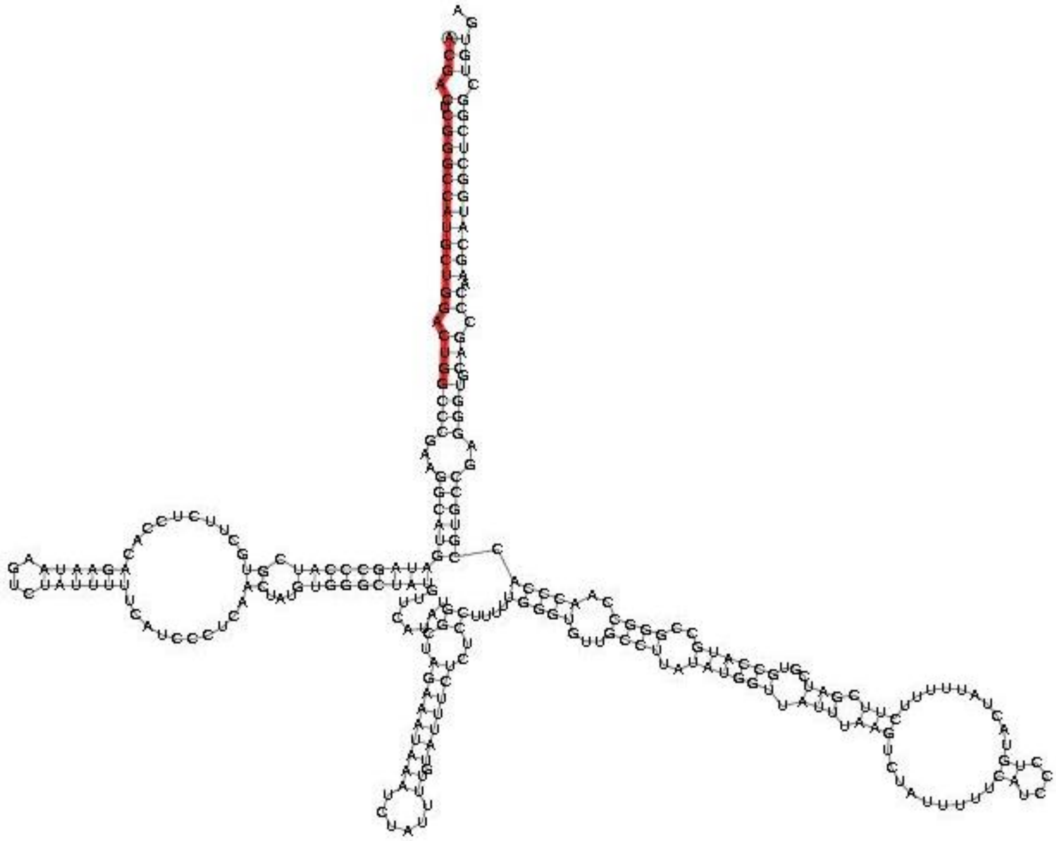

novel_157

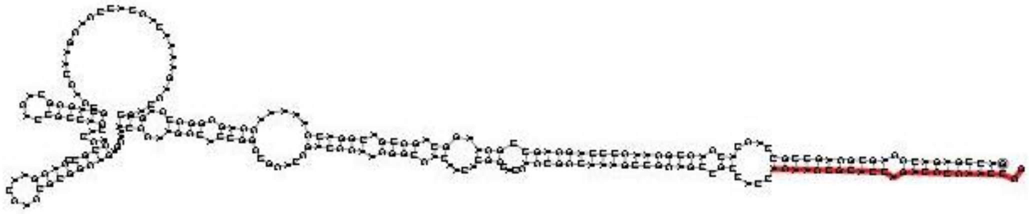

novel_158

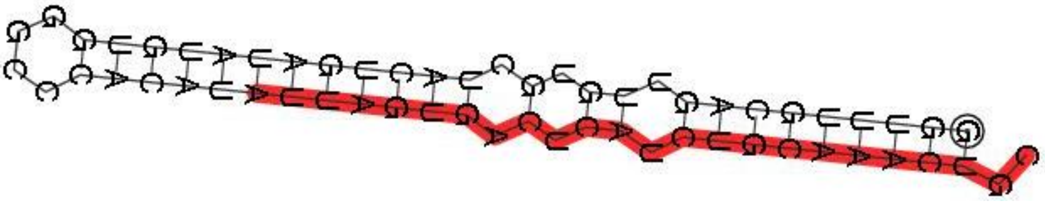

novel_159

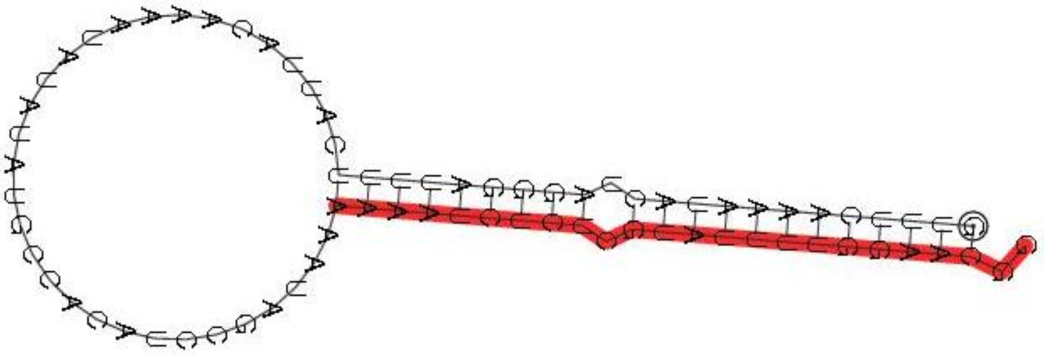

novel_163

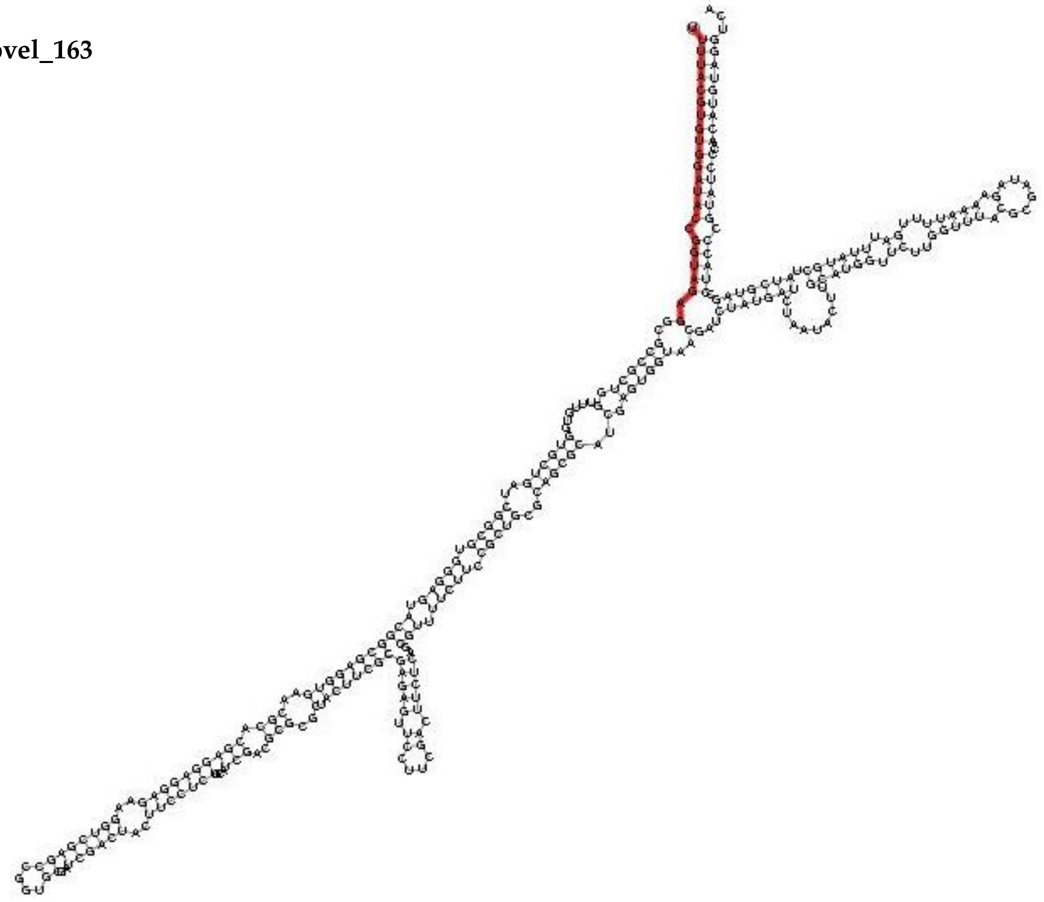

novel_165

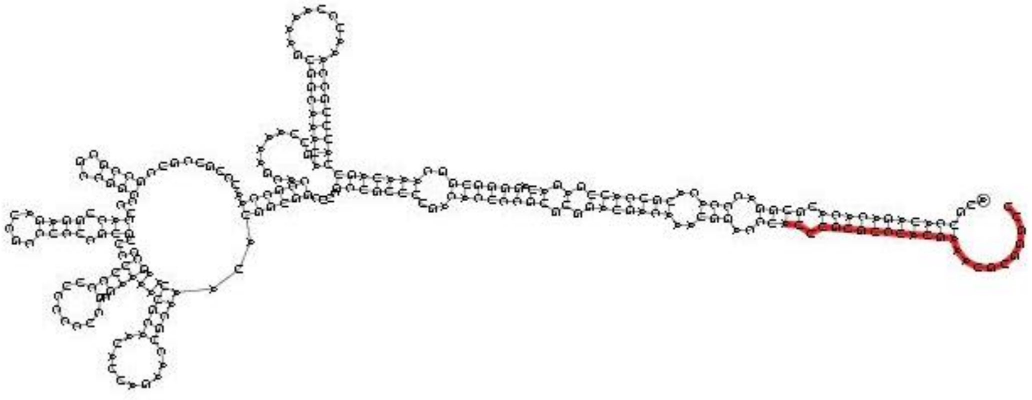

novel_171

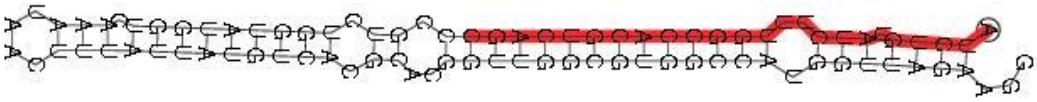

novel_187

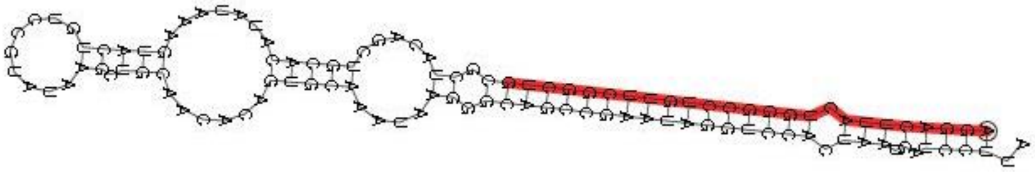

novel_193

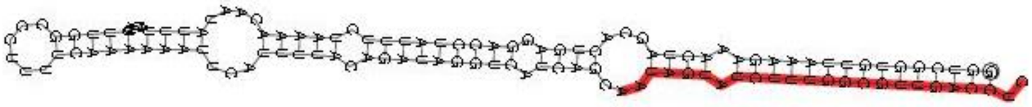

novel_198

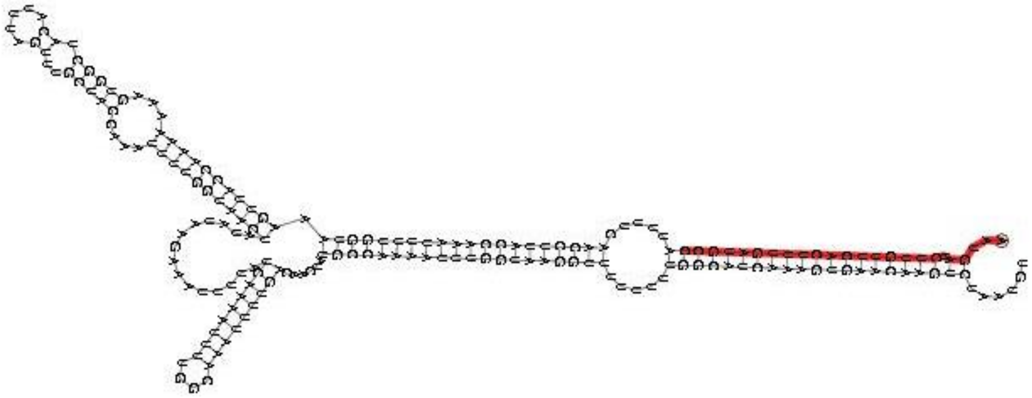

novel_199

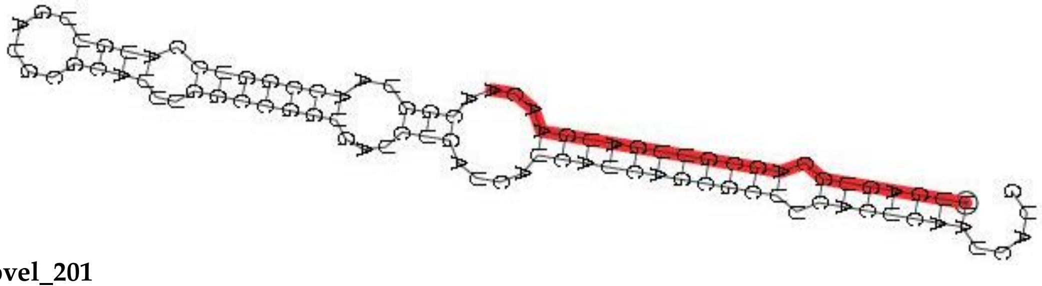

novel_201

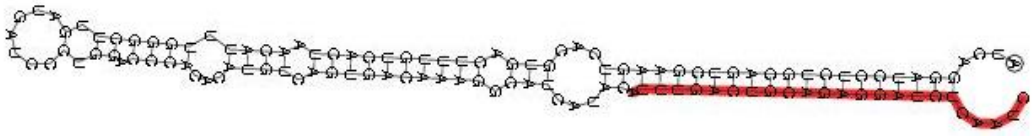

novel_202

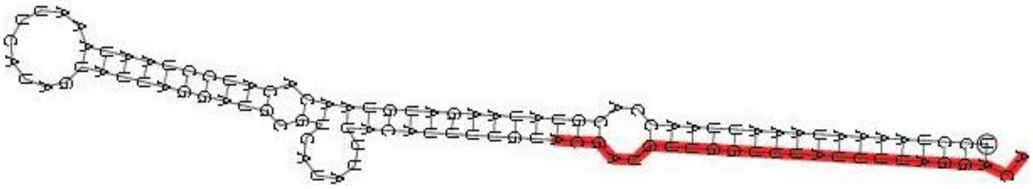

novel_203

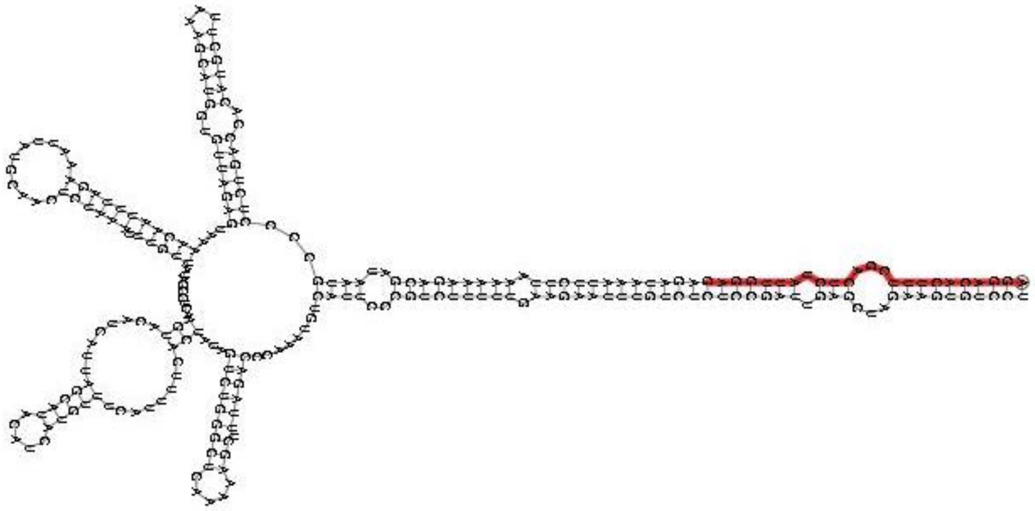

novel_209

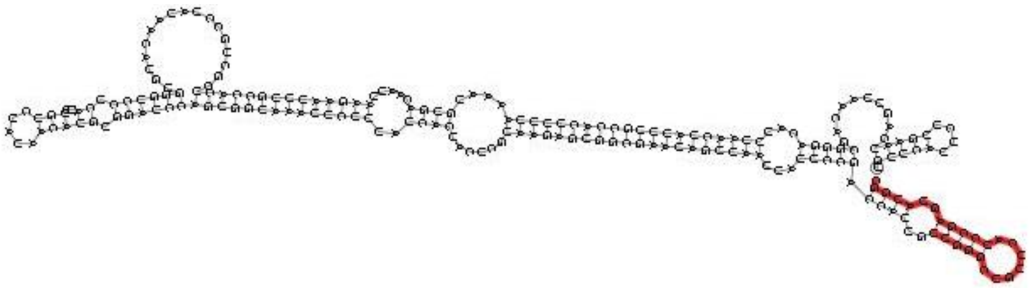

novel_210

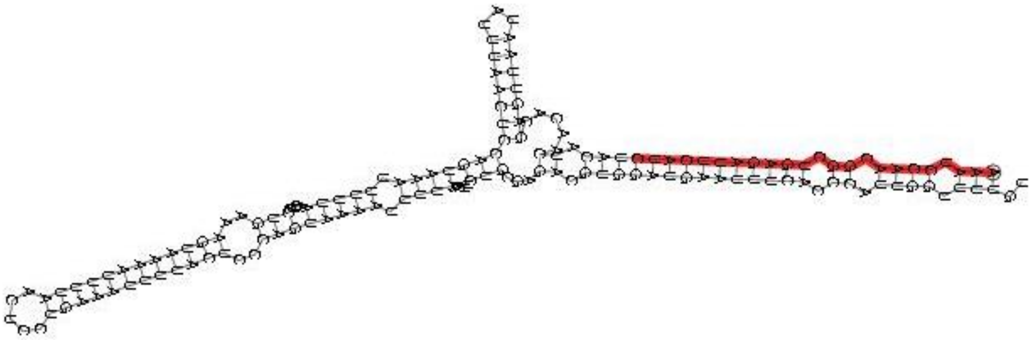

novel_213

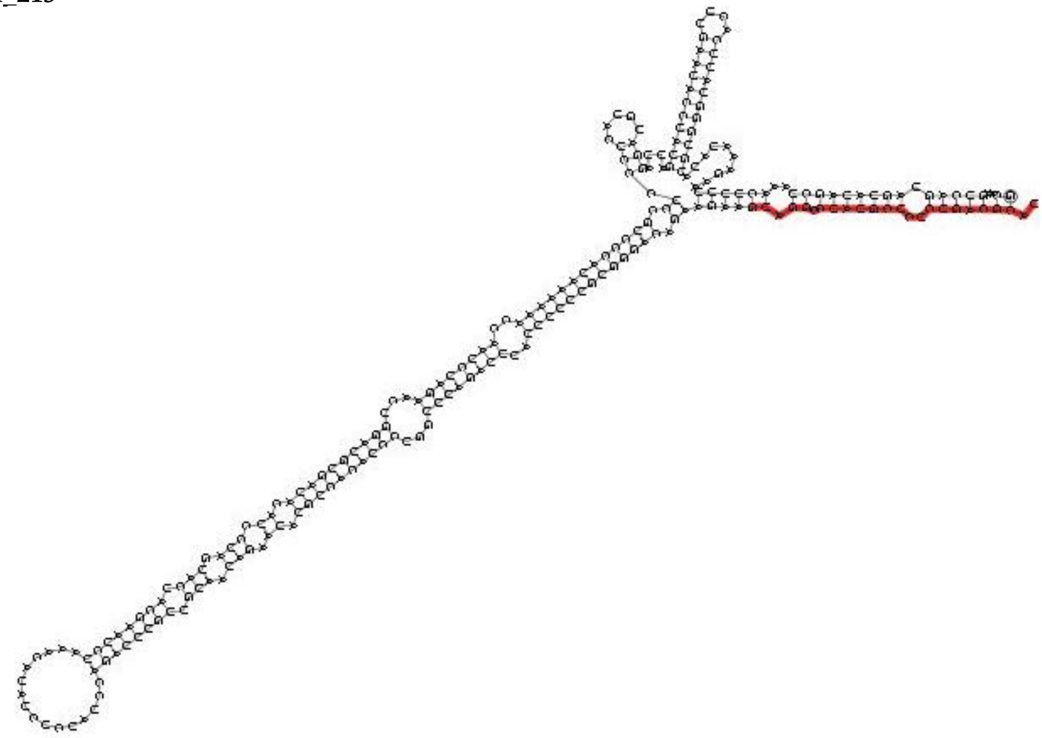

novel_223

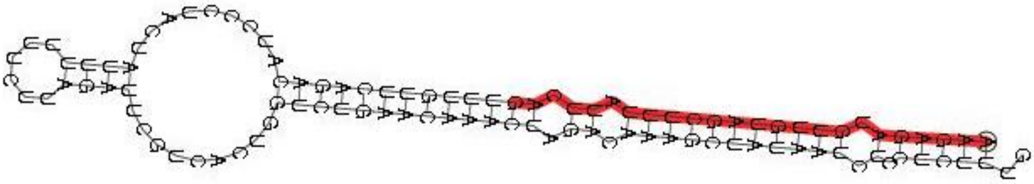

novel_236

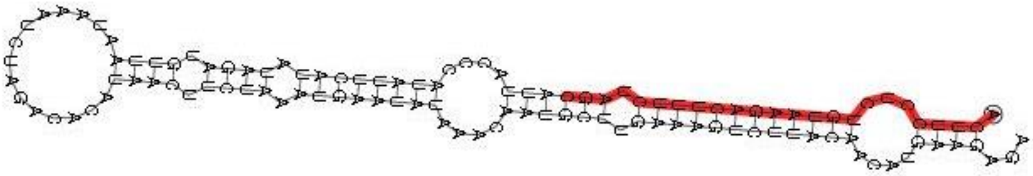

novel_241

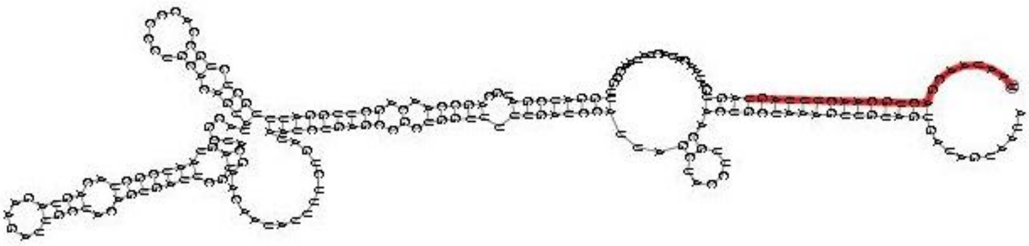

novel_242

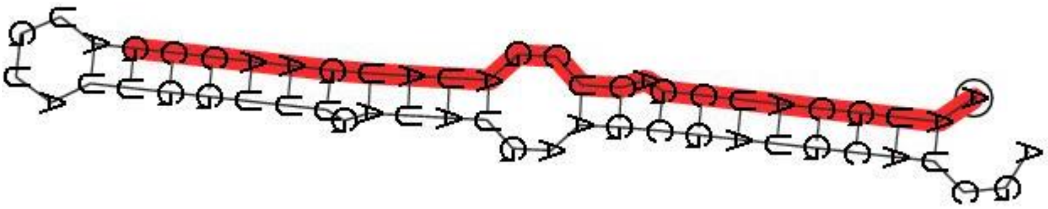

novel_244

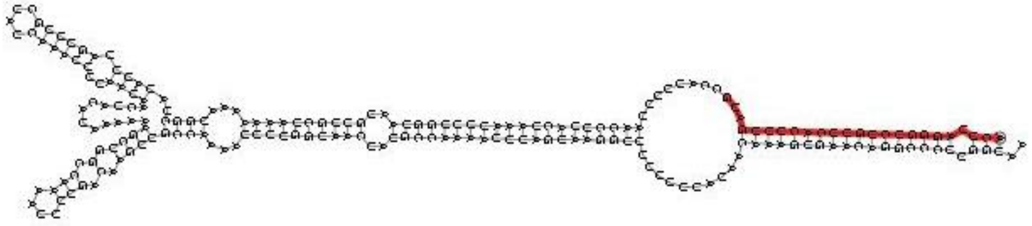

novel_248

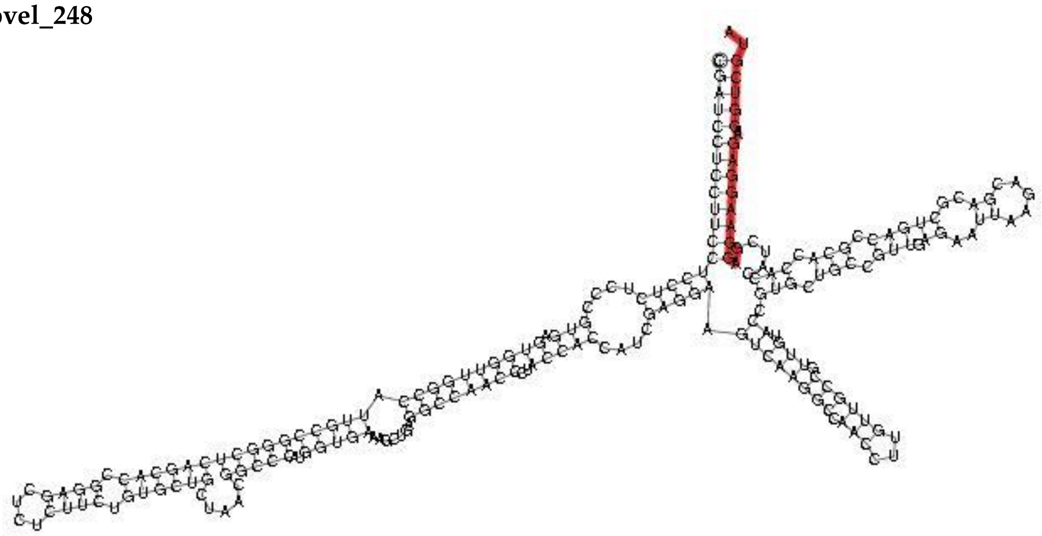

novel_251

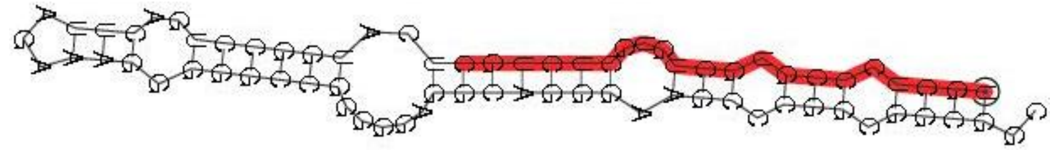

novel_253

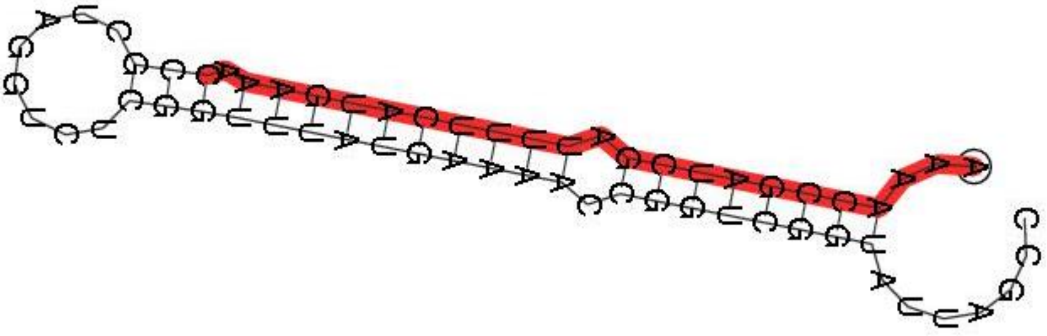

novel_254

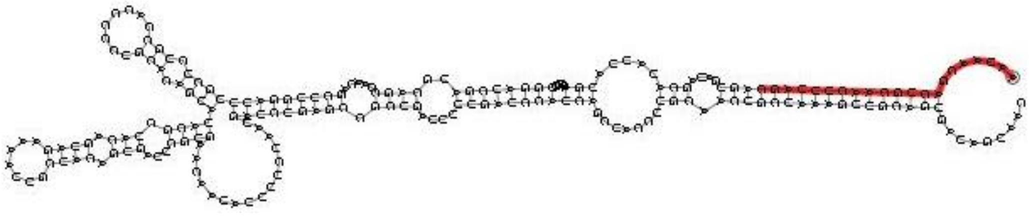

novel_255

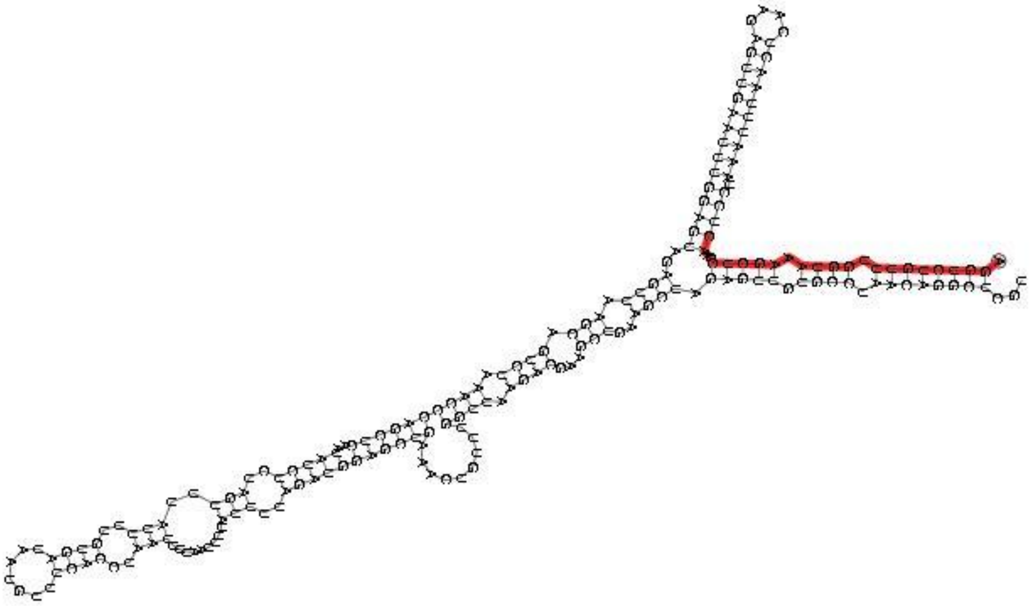

novel_256

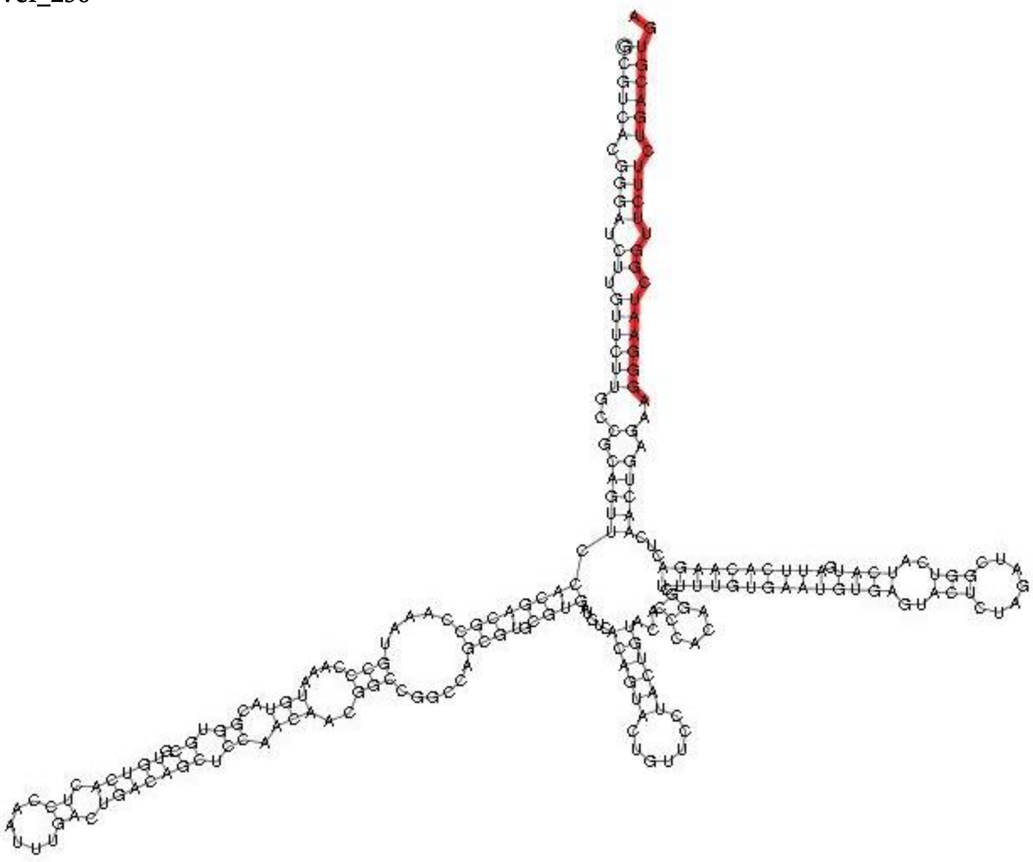

novel_263

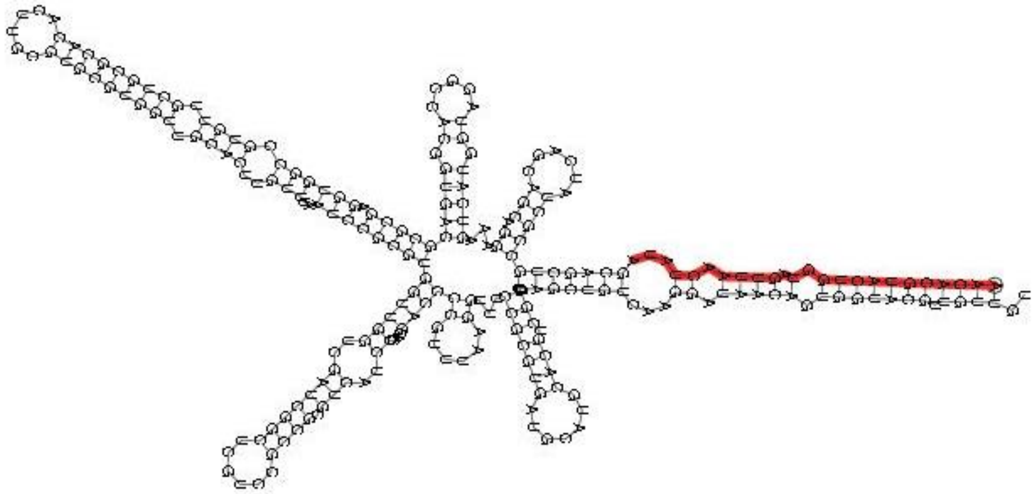

novel_264

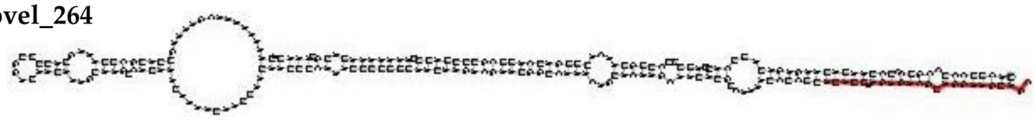

novel_265

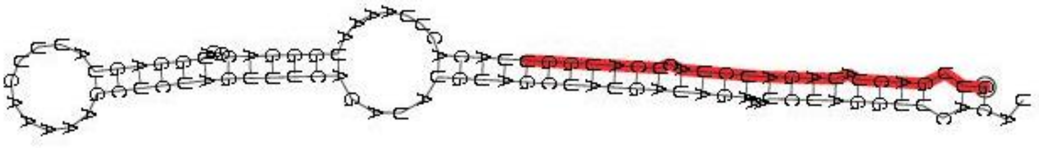

novel_266

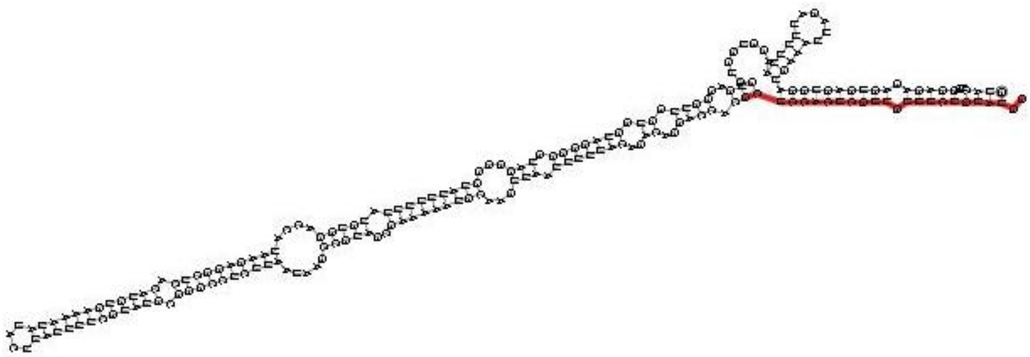

novel_268

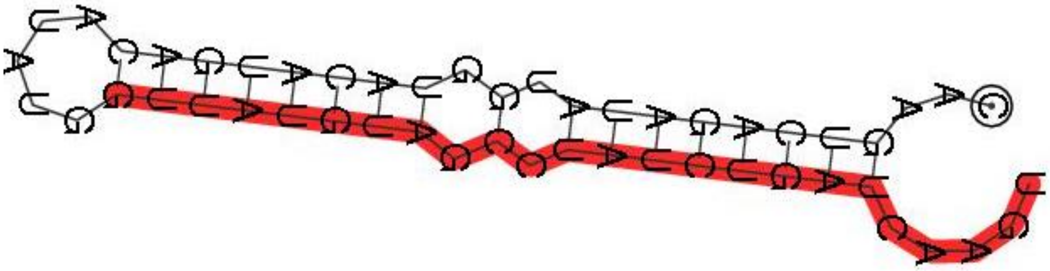

novel_270

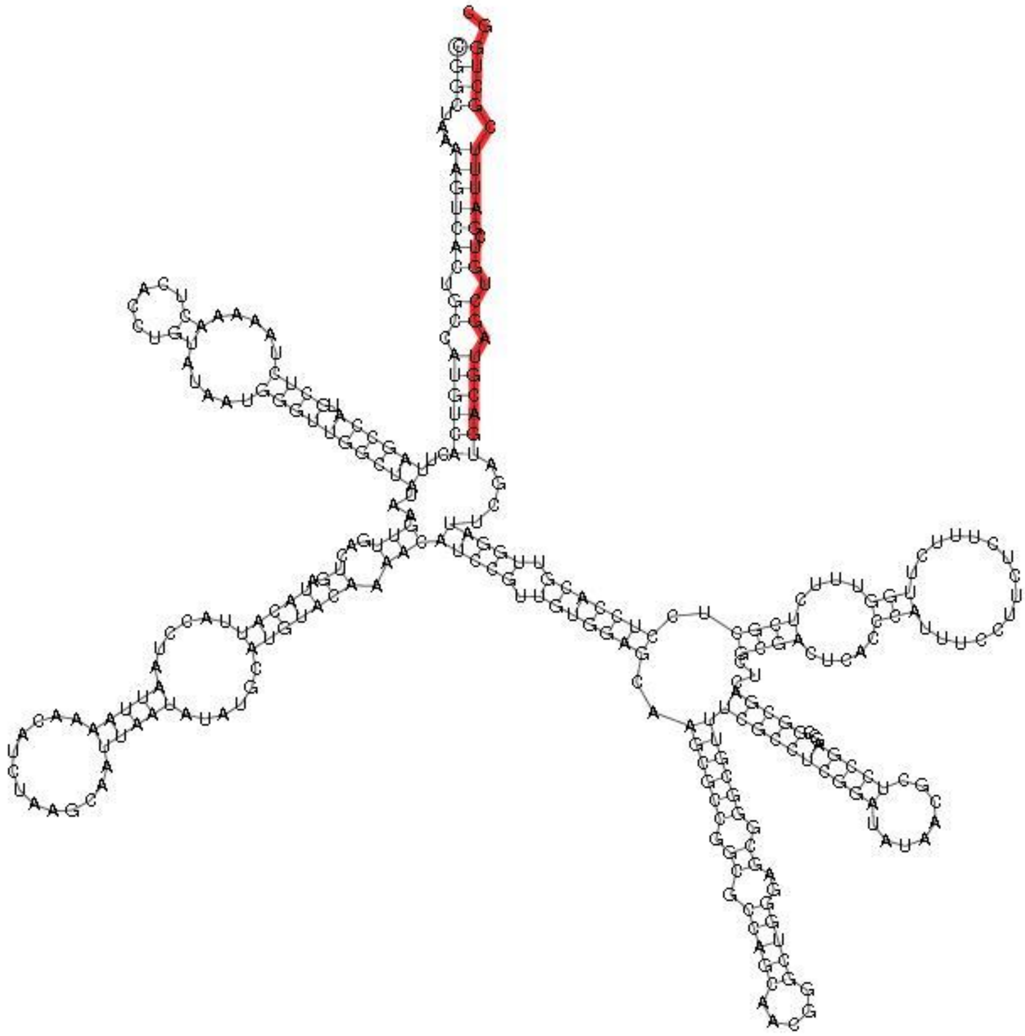

Supplement: Supplementary file 1 [file ijms-22-09958-s001.zip › Figure S1.pdf]
