# Supplementary material for: Deep Sequencing Discovery and Profiling of Known and Novel miRNAs Produced in Response to DNA Damage in Rice
Source: Int J Mol Sci. 2021 Sep 15;22(18):9958. doi: 10.3390/ijms22189958 (PMC8472271; doi:10.3390/ijms22189958)
Supplement: Supplementary file 1 [file ijms-22-09958-s001.zip › Figure S2.pdf]

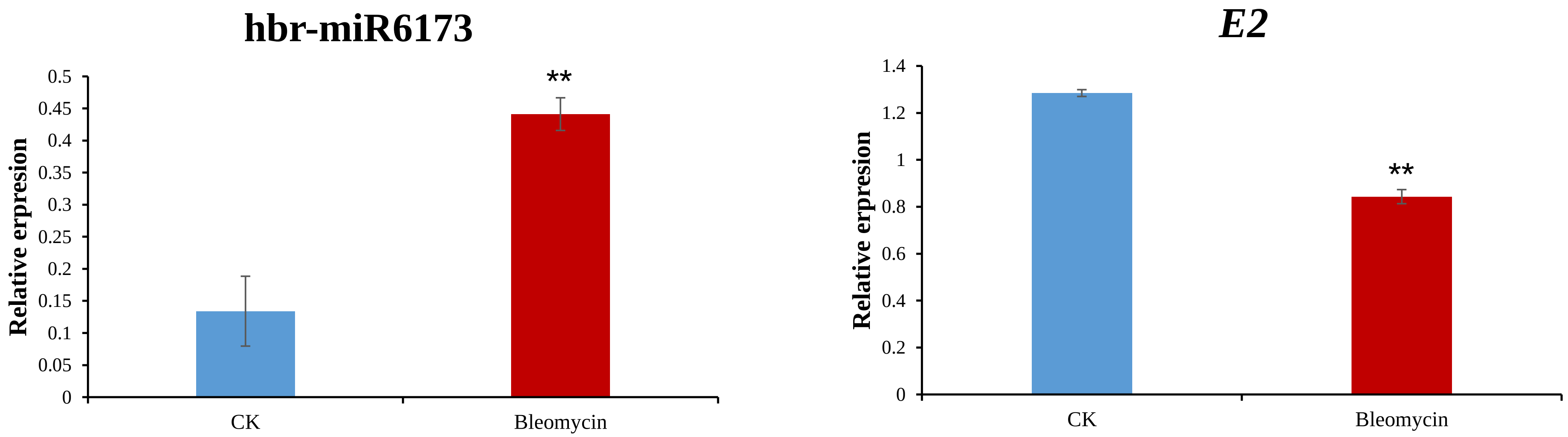

Figure S2. Verification of expression levels of hbr-miR6173 and its target gene *E2* by qRT-PCR.
U6 rRNA and *UBQ* (ubiquitin gene) gene were used as the internal control for miRNA expression and targeted genes expression respectively. Each bar indicates the mean ± SE of triplicate assays. ** indicate significant differences at P < 0.01 using fisher's protected least significant difference (PLSD) tests.
